# Dynamic ensemble prediction of cognitive performance in spaceflight

**DOI:** 10.1038/s41598-022-14456-8

**Published:** 2022-06-30

**Authors:** Danni Tu, Mathias Basner, Michael G. Smith, E. Spencer Williams, Valerie E. Ryder, Amelia A. Romoser, Adrian Ecker, Daniel Aeschbach, Alexander C. Stahn, Christopher W. Jones, Kia Howard, Marc Kaizi-Lutu, David F. Dinges, Haochang Shou

**Affiliations:** 1 Department of Biostatistics, Epidemiology, and Informatics, University of Pennsylvania Perelman School of Medicine, 219 Blockley Hall, 423 Guardian Drive, Philadelphia, PA 19104 USA; 2 Unit for Experimental Psychiatry, Division of Sleep and Chronobiology, Department of Psychiatry, University of Pennsylvania Perelman School of Medicine, 423 Guardian Drive, Philadelphia, PA 19104 USA; 3 Toxicology and Environmental Chemistry, National Aeronautics and Space Administration, 2101 E NASA Pkwy, Houston, TX 77058 USA; 4 Center for Toxicology and Environmental Health LLC, 2000 Anders Ln, Kemah, TX 77565 USA; 5 Department of Sleep and Human Factors Research, Institute of Aerospace Medicine, German Aerospace Center, Linder Höhe, 51147 Cologne, Germany; 6 Institute of Experimental Epileptology and Cognition Research, Faculty of Medicine, University of Bonn, Building 076, Venusberg-Campus 1, 53127 Bonn, Germany

**Keywords:** Human behaviour, Statistical methods

## Abstract

During spaceflight, astronauts face a unique set of stressors, including microgravity, isolation, and confinement, as well as environmental and operational hazards. These factors can negatively impact sleep, alertness, and neurobehavioral performance, all of which are critical to mission success. In this paper, we predict neurobehavioral performance over the course of a 6-month mission aboard the International Space Station (ISS), using ISS environmental data as well as self-reported and cognitive data collected longitudinally from 24 astronauts. Neurobehavioral performance was repeatedly assessed via a 3-min Psychomotor Vigilance Test (PVT-B) that is highly sensitive to the effects of sleep deprivation. To relate PVT-B performance to time-varying and discordantly-measured environmental, operational, and psychological covariates, we propose an ensemble prediction model comprising of linear mixed effects, random forest, and functional concurrent models. An extensive cross-validation procedure reveals that this ensemble outperforms any one of its components alone. We also identify the most important predictors of PVT-B performance, which include an individual's previous PVT-B performance, reported fatigue and stress, and temperature and radiation dose. This method is broadly applicable to settings where the main goal is accurate, individualized prediction of human behavior involving a mixture of person-level traits and irregularly measured time series.

## Introduction

Space travel is a costly and hazardous endeavor. Astronauts are often faced with cognitively demanding tasks that require sustained attention, despite chronic sleep deprivation and disruptions to their circadian rhythms^1^. Human performance deteriorates without proper sleep, manifesting in slower reaction times and increased errors^2^, heightening the risk of operational accidents^3^. Therefore, it is critical to anticipate changes in alertness and performance on a dynamic and individualized basis^4^. Vigilant attention is a construct typically assessed using reaction time and accuracy-based metrics in tasks requiring sustained attention. While environmental and psychological correlates of vigilant attention have been studied in healthy humans on Earth^5,6^, highly trained and carefully selected astronauts are not necessarily represented in this population. Astronauts are also exposed to a unique set of conditions in space^7–9^, including microgravity, extended confinement and isolation, radiation exposure, and other environmental and operational extremes. The collective impact of these challenges on psychological health and performance is inconclusive^10,11^ and not yet fully understood^12,13^.

The goal of this study was to dynamically predict vigilant attention, assessed with a brief 3-min version of the Psychomotor Vigilance Test (PVT-B)^14^, as a function of astronauts' past performance, self-reported stress and fatigue, demographic and operational information, and variations in environmental variables (Table S1). The main challenge to predicting PVT-B performance is unraveling variation associated with the circadian rhythm, individual traits, psychological state, and the external environment^15,16^. Previously, PVT performance was predicted via a two-process model^17^, which incorporates a system of differential equations to describe homeostatic and circadian pressures governing sleep. While such models have expanded our understanding of sleep regulation and alertness, and have been greatly adapted^18–20^, they are often deterministic and so preclude statistical comparisons; even models with person-level random effects^21^ cannot typically accommodate a large number of covariates.

Statistical models offer a complementary approach to prediction, focusing on prediction accuracy and uncertainty estimation at the expense of only indirectly modelling physiological processes. Traditional methods for assessing the associations between PVT performance and sleep patterns have included correlation and ANOVA analyses^22,23^, which allow for hypothesis testing but cannot make forecasts of later performance, adjust for the autocorrelation in repeated PVT measures over time, or accommodate the non-linear relationships between PVT performance and predictors^24^. Methods which have addressed these obstacles have mainly considered mixed-effect models^25^ or an ensemble of mixed-effects and random forest models^26^. However, neither of these methods can explicitly model time-varying predictors whose effects themselves are time-varying, as in the case of circadian effects^27^ or acclimation^28,29^.

In this paper, we propose a 3-model ensemble prediction scheme consisting of a linear mixed effects model^30^, a random forest model^31^, and a functional concurrent model^32^, the last of which allows us to estimate time-varying effects of each (potentially time-varying) predictor. We also incorporate predicted outcomes from a two-process model^18^ as a covariate, with the aim of connecting biomathematical and statistical models commonly used to predict PVT performance. Our method extends the 2-model ensemble proposed by Cochrane and colleagues^26^, though we employ a variant of forward-chaining cross-validation^33^ to assess model performance. We demonstrate that the ensemble best predicts over the entire mission compared to any single component alone.

## Material and methods

### Participants and protocol

Reaction Self-Test (RST; see “Reaction self-test (RST)” section) data were collected from N = 24 astronauts (Table 1) over 19 International Space Station (ISS) mission increments between 2009 and 2014^34^. Astronauts spent an average of 160 (SD = 19) days, with a range of 123–192 days, on the ISS. Two versions of the RST were used: a morning version was taken after awakening from sleep, and an evening version prior to bed. Ahead of spaceflight, astronauts were scheduled to complete the RST twice per testing day (i.e., one morning RST and one evening RST per day) at 180, 120, 90, 60, and 30 days before launch and daily in the week before launch. Post-mission RST assessments were scheduled daily in the week after return to Earth as well as once at 30, 60, and 90 days after return. During the space mission, astronauts were instructed to complete the RST twice a day every 4 days, with extra sessions completed around extravehicular activities (EVAs) and sleep period shifts to accommodate spacecraft dockings. The total adherence rate of 78.9% across all RSTs (83.8% in-flight) exceeded the pre-determined project goal of 75% adherence. This resulted in a total of 2968 RST observations. The original study and this retrospective analysis were approved by the Institutional Review Boards of Johnson Space Center and the University of Pennsylvania (for data analysis); all research was performed in accordance with relevant regulations and guidelines. Participants provided written informed consent prior to study participation and re-consented for this retrospective analysis.

**Table 1:** Summary characteristics of the astronauts with reaction self-test (RST) data.

	(N = 24)
**Sex, n (%)**	
Male	19 (79.2)
Female	5 (20.8)
Age at dock, years	48.2 (4.78)
Prior days in space	53.5 (72.7)
Prior missions	1.29 (0.86)
**Highest educational attainment, n (%)**	
Master's	14 (58.3)
MD/PhD	10 (41.7)
**Nationality/agency, n (%)**	
USA/NASA	16 (66.7)
Non-USA/non-NASA	8 (33.3)
Average pre-flight overall performance score (OPS)	0.95 (0.02)
Number of in-flight RST observations	87.2 (18.8)

Table values are mean (standard deviation) and count (percent) for continuous and categorical variables, respectively. Due to astronaut privacy concerns, marital status and number of children are not reported in this table.

### Reaction Self-Test (RST)

The RST consists of a short survey (described below) followed by a computerized and brief (3-min) version of the Psychomotor Vigilance Test (PVT-B). The PVT is a validated measure of sustained attention based on reaction time (RT) to visual stimuli that occur at random inter-stimulus intervals^35^. Astronauts were instructed to monitor a box on the laptop screen and press the space bar once a millisecond counter appeared in the box and started incrementing. After that, the RT was displayed for 1 s and the next stimulus was presented after a random inter-stimulus interval of 2-5 s. Participants were instructed to react as quickly as possible without hitting the spacebar in the absence of a stimulus. The PVT-B has been recognized as a sensitive tool for detecting the effects of acute and chronic sleep deprivation and circadian misalignment, both of which are highly prevalent in spaceflight^1,36^. It has negligible aptitude and learning effects^37^, and is ecologically relevant as sustained attention deficits and slow reaction times affect many real-world tasks, including the operation of a moving vehicle^2^.

Astronauts were instructed to perform the RST in the morning after getting up and in the evening in the 2 h prior to bed, though the hour of the day varied (Figure S1). While the PVT-B portion is the same in both morning and evening versions, other portions differ slightly. The survey portion of the RST includes a sleep diary and 11-point Likert-type rating scales on tiredness, mental fatigue, physical exhaustion, stress, sleepiness, and a final rating depending on the time of day: workload (evening administration only) or sleep quality (morning administration only; Table S2). During both the morning and evening RSTs, astronauts were asked to list the name, dose unit, and doses taken of all medications ingested before going to bed the previous night (morning RST) and since awakening in the morning (evening RST). Additionally, in the evening RST, astronauts were asked to list caffeinated foods or beverages consumed since awakening in the morning (in both cases, “None” and “Decline to answer” were response alternatives). Astronauts were also asked whether they performed an EVA that day. This information was used to create binary variables for certain classes of medications and upcoming EVAs for each RST observation (Table 2).

**Table 2:** Summary measures from the reaction self-test (RST) data, including pre- and post-flight observations.

Total number of observations	(N = 2968)
**Period, n (%)**	
Pre-flight	506 (17.0)
In-flight	2109 (71.1)
Post-flight	353 (11.9)
**Time of day, n (%)**	
Morning	1568 (52.8)
Evening	1379 (46.5)
Other	21 (0.71)
**Alertness**	
LRM-50	− 33.0 (12.7)
**Sleep**	
Time in bed sleeping, hours	6.61 (1.30)
Time in bed not sleeping, hours	0.61 (0.77)
**Self-report 11-point ratings**	
Low workload (0–10)	4.47 (2.18)
Very stressed (0–10)	3.87 (2.01)
Poor sleep quality (0–10)	3.60 (1.87)
**Medication use**	
Caffeine, doses	2.05 (1.50)
Sleep aid flag, n (%)	131 (4.41)
Decongestant flag, n (%)	25 (0.84)
Antihistamine flag, n (%)	36 (1.21)
Pain medication flag, n (%)	143 (4.82)
**Extravehicular activity (EVA)**	
EVA today flag, n (%)	8 (0.27)
EVA tomorrow flag, n (%)	23 (0.77)

Table values are mean (standard deviation) and count (percent) for continuous and categorical variables, respectively.

**Figure 1:**
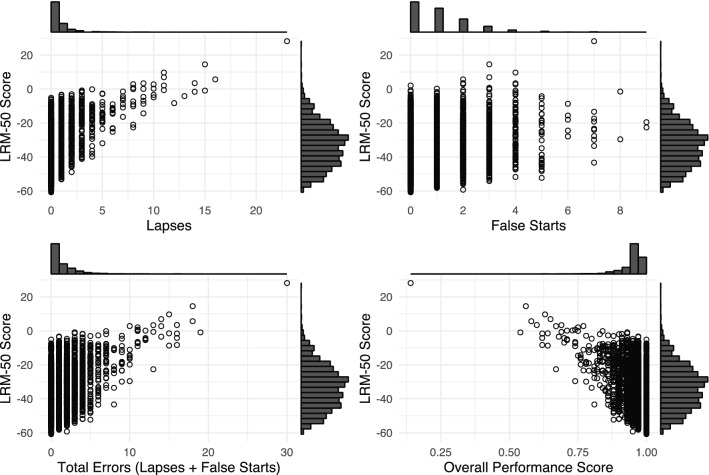
The LRM-50 score is best able to differentiate between the high performers in our sample, which includes pre-, post-, and in-flight observations (n = 2968) for all astronauts. These scatterplots show the joint distribution of LRM-50 with four popular PVT metrics: lapses, false starts, total errors (lapses + false starts), and the overall performance score (OPS). The histograms at the top and right edges show the marginal distributions of the variables on the x- and y-axes, respectively. Compared to other PVT metrics, the LRM-50 score is more normally distributed and is more sensitive to better performers (i.e., those with lower LRM-50).

Among the PVT-B performance metrics, we derived the LRM-50 as the outcome of interest, since it has been shown to be highly sensitive to sleep deprivation and has an approximately normal distribution^38^. LRM-50 is a likelihood ratio-based metric that is based on response time (RT) distributions derived from either a non-sleep deprived (non-SDP) state corresponding to the first 15 h of wakefulness, or a sleep deprived (SDP) state corresponding to hours 15 through 33 of wakefulness. In the original study, these distributions were derived from participants in a total sleep deprivation protocol who performed the PVT every 2 h^38^. The RT space was divided into 50 categories consisting of 49 RT intervals plus false starts. For a certain RT range, the likelihood ratio was calculated as the relative frequency of responses falling into the range under the SDP condition, divided by that of responses falling into the range under the non-SDP condition. Likelihood ratios greater than 1 indicate that responses in that range are more likely to be observed under the SDP condition compared to non-SDP, and conversely for likelihood ratios less than 1. The LRM-50 score is calculated by determining the RT range and associated likelihood ratio for each PVT-B stimulus. Likelihood ratios of all stimuli are then multiplied and log-transformed to induce symmetry around zero. Therefore, an LRM-50 of 0 means that this test bout is equally likely to be observed under an SDP or non-SDP condition. When LRM-50 < 0, the non-SDP condition is more likely relative to SDP, and conversely for LRM-50 > 0. LRM-50 correlates highly with response speed (reciprocal RT), but has the advantage that it also takes false starts (i.e., premature responses) into account. Compared to LRM-50, other commonly used PVT metrics^35^, such as the number of lapses or false starts, were less effective in differentiating high performers such as astronauts (Fig. 1). We also considered a standardized measure of LRM-50, linearly scaled by each participant's mean and standard deviation.

Finally, as a person-level measure of baseline performance, we considered each participant's average pre-flight Overall Performance Score (OPS):

$${\text{Overall Performance Score}} = 1 - \frac{{{\text{False Starts}} + {\text{Lapses}}}}{{{\text{Valid Stimuli }}\left( {\text{Including False Starts}} \right)}}.$$

The OPS is moderately sensitive to sleep loss, combines false starts and lapses into a single number, and is easily interpretable: an OPS of 1 corresponds to perfect performance, while 0 corresponds to the worst possible performance.

### Environmental data

During the in-flight study period, five domains of environmental measures were recorded on the ISS. Radiation dose levels were obtained from the Space Radiation Analysis Group at NASA Johnson Space Center, and were summarized in daily absorbed dosage units (mGy) based on readings from dosimeters located aboard the ISS. The radiation dose was defined as the sum of radiation due to Galactic Cosmic Rays and the South Atlantic Anomaly. Measurements were collected from the following instruments over the course of the study: passive dosimeters (Radiation Area Monitor, 2009–2012), active dosimeters (Radiation Environment Monitor, 2012–2014), Tissue Equivalent Proportional Counter (TEPC, 2009–2012), and Intravehicular Tissue Equivalent Proportional Counter (IV-TEPC, 2013–2014). These instruments were rotated between several ISS modules (US Lab, Node 2, JEM, Columbus Module, and Service Module) at different times during the study period (Fig. S2).

Next, oxygen (O_2_) and carbon dioxide (CO_2_) levels in units of mmHg were collected from Major Constituent Analyzer (MCA) sample inlet ports located throughout the space station's air circulation system (Fig. S2, Panel B). Samples were drawn from these inlet ports in a cyclical fashion and were analyzed in two mass spectrometer-based MCA units located in Node 3 and the US Lab. These units alternated between being the primary or backup unit, ensuring redundancy during scheduled maintenance or malfunction. We used the reading from whichever served as the primary sensor at the time. Temperature in Celsius (°C) was measured by sensors in the Node 2, Node 3, and US Lab modules. Temperature, CO_2_, and O_2_ data was downloaded from the Java Mission Evaluation Workstation System in intervals of at least 1 reading per second.

Noise exposure in A-weighted decibels (dBA) was not continuously monitored, but was rather collected periodically. Astronauts set up acoustic dosimeters at rotating locations aboard the ISS for 24-h periods approximately every other month. The internal memory of these dosimeters allowed the recording of dBA levels in one-minute intervals, which were acquired through manual display recall and infrared serial interface download.

### Demographic and operational data

Demographic information was obtained from all astronauts (Table 1) and included sex, age at the time of ISS docking, nationality, space agency, educational attainment, number of prior space missions, and prior days in space. Operational data included the number of occupants on the ISS for each day of the mission and proximity of test to dock/undock maneuvers or EVAs.

### Derived predictors

We derived several predictors based on the RST data: a stress/fatigue composite score, four medication flags, and predicted PVT lapses given the sleep schedule. The stress/fatigue composite score was created based on principal components analysis (PCA) of the 11-point scales on which the crew rated several behavioral states (i.e., sleepiness, tiredness, fatigue, exhaustion, stress, workload, and sleep quality) before taking the PVT. The score was calculated as the weighted average of the 11-point rating questions, with weights determined by the loadings onto the first principal component (PC). Table S3 shows the loadings onto the first PC, which accounted for 48.3% of the variance. Higher values of the stress/fatigue composite variable correspond to increased tiredness, more stress, and worse sleep quality. The loading for workload was negligible, potentially due to its lack of correlation with the other variables. Next, medication use was coded as a binary variable for four broad categories: pain medications, sleep aids, decongestants, and antihistamines. These categories were chosen due to their established use by astronauts on the ISS^39^ and because their use may affect sleep or be correlated with conditions affecting sleep or alertness^40–42^. To represent the complex information contained in the sleep schedule, the final derived covariate was the number of predicted PVT lapses under a two-process model^18^, which was calculated solely using an individual's reported bedtime and wake time.

### Data integration and interpolation

To integrate environmental data with RST data, several strategies were required as different variables were recorded at different time intervals: RST was collected twice a day every 4 days; radiation dose and other operational variables were measured daily; temperature, noise level, CO_2_, and O_2_ were measured multiple times per day or minute (Table S4). For each RST observation, the value of radiation and ISS occupancy from that day was used. For temperature, noise, CO_2_, and O_2_, we used the average during the hour that the RST was completed, if available. Due to the logarithmic nature of decibel units, noise values were always averaged using the energetic average, while all other variables were averaged using the usual arithmetic mean.

During some 1-h periods, noise and temperature measurements were available for more than 1 sensor. For RST observations that occurred during these times, we employed location matching to achieve the best estimate for that individual. When the RST was taken on a computer located in Node 2 (where the crew quarters are located) or the US Lab, only the temperature or noise data from the corresponding Node 2 or US Lab was used. When the RST was taken elsewhere or the location was unknown, a weighted average of the Node 2 (75%) and US Lab (25%) measurements were used, reflecting the approximate empirical frequency of RSTs taken in these modules. Other variables (CO_2_, O_2_, and radiation) were only measured from one sensor at a time, so they were not location-matched.

When the daily or hourly value of an environmental variable was unavailable, we used two interpolation strategies, which are summarized in Table S4. Temperature, O_2_, and CO_2_ data had a relatively low rate of missingness (Table S5), so the locally estimated scatterplot smoothing (LOESS, neighborhood parameter $\alpha$ = 0.1) value was used when the hourly average was not observed. Enough temperature data was available for Node 2 and US Lab that we could fit 3 separate LOESS interpolations: one for RST observations in Node 2, one for US Lab, and one for other or unknown locations which were interpolated using the weighted average described previously.

**Figure 2:**
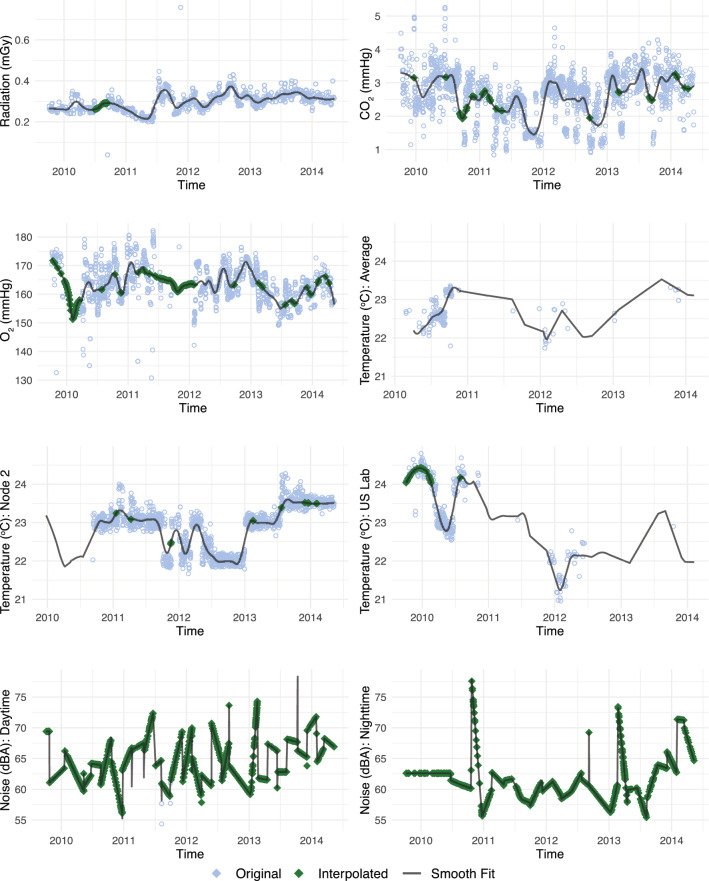
For each RST observation, the corresponding value of the environmental variable was found by using the observed value (if available) or the interpolated value formed by neighboring observations (“Data integration and interpolation” section). These plots illustrate the LOESS curves (black line) fit to the entire environmental data for radiation, temperature (separately for each location), CO_2_, and O_2_. A linear interpolation was used for noise (separately for daytime and nighttime). Each hollow blue circle corresponds to the observed hourly average (CO_2_, O_2_, temperature) or daily average (noise, radiation) that was used for an RST observation; the green diamond indicates that the interpolated value was used.

Noise levels were recorded on only 47 occasions throughout the study period, for a total of 722 hourly values. However, RST observations only rarely coincided with days that the acoustic dosimeters were active; as a result, 99.71% of in-flight RST observations did not have an observed noise level. For interpolation, we assumed that noise levels followed a 24-h cycle that was similar between days, with higher noise levels during the daytime (defined as 7:00 AM to 10:59 PM UTC) compared to nighttime (11:00 PM to 6:59 AM UTC) (Figure S3). Then, noise levels were interpolated using separate linear interpolations for averaged daytime and nighttime values. In other words, the noise level for a daytime RST observation was the interpolated value between the average daytime noise level from the most recent day of noise collection, and the next upcoming day; the same procedure was used for nighttime noise. The distribution of smoothed and unsmoothed environmental data is shown in Fig. 2.

Finally, the predicted PVT lapses depended solely on each individual's reported sleep schedule consisting of bedtimes and wake times. For RST observations where the sleep time was not reported, these variables were carried forward from the last observation for that individual, and the predicted lapses were calculated using the observed and interpolated bedtimes and wake times. One individual did not report any sleep data, so their predicted lapses were replaced by the overall average.

### Statistical models

Our main goal was to construct a statistical model to predict the LRM-50 score for each participant at future points in time. Of the variables collected, we were also interested in identifying a subset of variables that were most important to predicting LRM-50. Candidate predictors of LRM-50 included a mixture of time-varying (i.e., function-valued) variables such as environmental data, most recent LRM-50 score, self-reported stress/fatigue score, and ISS occupancy, as well as person-level (i.e., scalar-valued) data including each participant's demographics, pre-flight average PVT, sex, and age at docking.

We employed an ensemble of several models to address each aspect of the data. For participant $i$ and time $t$, the linear mixed effects (LME) model defines the LRM-50 score $y_{it}$ as a function of $p$ covariates $X_{it} = \left( {X_{it}^{\left( 1 \right)} , \ldots , X_{it}^{\left( p \right)} } \right)$, intercept $\beta_{0}$, a $p$-dimensional vector of fixed effects $\beta ,$ person-specific random intercept $b_{i}$, and error $\varepsilon_{it}$:

$$y_{it} = \beta_{0} + X_{it} \beta + b_{i} + \varepsilon_{it.}$$

The advantages of LME include its simplicity and efficiency, as well as the option to model correlated measurements over time: we specified a lag-one autoregressive (AR1) correlation structure to model the repeated measures of $y_{it}$.

By contrast, the random forest model^31^ specifies no closed form for the relationship between $y_{it}$ and $X_{it}$; rather, it uses an aggregate of decision trees to identify splitting points for continuous variables that optimally predict the outcome. While prone to overfitting, random forests are able to model a more flexible non-linear relationship between outcome and predictors, at the cost of interpretability.

Finally, since neither the random forest nor the LME are able to model the serial dependence of time-varying predictors and their time-varying effects, we also considered the functional concurrent model^32^: for participant $i$ and observation $j$ at time $t_{ij}$, the time-varying outcomes $y_{ij}$ are related to $p$ covariates $X_{ij}^{\left( 1 \right)} , \ldots , X_{ij}^{\left( p \right)}$ through the following:

$$y_{ij} = \beta_{0} + f_{1} \left( {X_{ij}^{\left( 1 \right)} ,t_{ij} } \right) + \ldots + f_{p} \left( {X_{ij}^{\left( p \right)} ,t_{ij} } \right) + b_{i} \left( {t_{ij} } \right) + \varepsilon_{ij} ,$$

where $f_{i}$ are smooth functions approximated by thin plate splines, and $b_{i} \left( t \right)$ and $\varepsilon_{ij}$ are Gaussian processes representing person-level random trajectories and time-independent errors, respectively. The ensemble prediction was then constructed as the average of the predictions from the LME, random forest, and functional concurrent model. All data analyses were performed using R version 3.6.1^43^, employing the *nlme*, *randomForest*, and *fcr* packages for each model.

### Model validation

**Figure 3:**
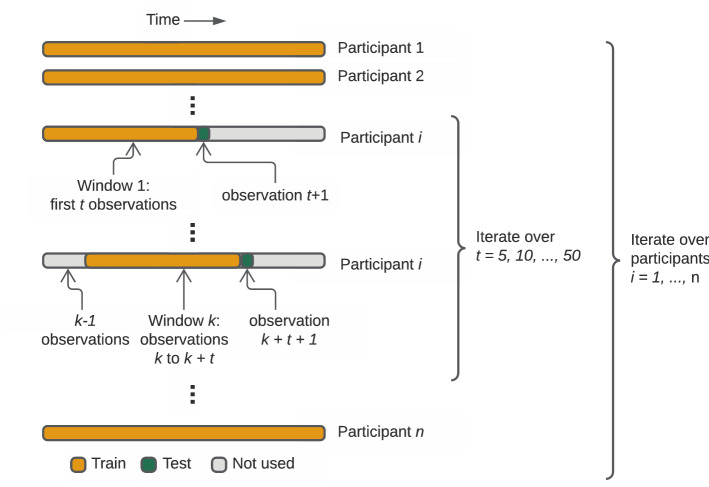
We used a forward-chaining procedure to assess prediction accuracy of a given model. For participant i and number of days t, the model was fit on window $k$ defined by the $k$ through ($k+t$)th Reaction Self-Test (RST) observations from participant $i$ and the full data for all other participants. Then, the model prediction on the subsequent day was compared to the observed value on that day. The person-level prediction accuracy for a given participant was defined as the averaged squared difference between predicted and observed values over all values of k. The overall accuracy was then defined as the average of the person-level prediction accuracies.

To assess the performance of each model as well as the ensemble, we employed a forward-chaining validation procedure (Fig. 3). For participant $i$ with $n_{i}$ RST observations, training length $t \in \left\{ {5, 10, \ldots , 45, 50} \right\}$, and window number $k \in \left\{ {1, 2, \ldots ,{\text{min}}\left( {20, n_{i} - t + 1} \right)} \right\},$ a given model was fit on window $k$ defined by the $k$ through ($k + t$)th RST observations from participant $i$ and all RST observations from all other participants. That is, the model is trained on a partial time series (window $k$) for person $i$ plus all other individuals' full time series. Then, the squared prediction error was assessed at both observation $k + t + 1$ (test) and window $k$ (training). By incrementing $t$, the size of the training set is allowed to increase; by incrementing $k$, the training set shifts in time, so that the model is trained and evaluated at different portions of the mission. In contrast to methods which define the training set as *all* points prior to observation $t + k + 1$^26^, controlling $t$ allows us to minimize biases due to individual heterogeneity in observation frequency and number.

To aggregate these point-level errors into an overall error metric over all individuals, we defined a metric that weighted individuals equally, despite variation in $n_{i}$. For a given number of training days $t$, prediction error in the test set was measured at several levels. First, we defined the $i$th person's error by the average squared error at day $t + k + 1$ (i.e., the test set) over all windows $k$:

$$MSE\left( {i,t} \right) = \frac{1}{{N_{i,t} }}\mathop \sum \limits_{k = 1}^{{N_{i,t} }} \left( {y_{itk} - \hat{y}_{itk} } \right)^{2} ,$$

where $y_{itk}$ is the true LRM-50 at day $t + k + 1$, $\hat{y}_{itk}$ is the predicted value, and $N_{i,t} = {\text{min}}\left( {20, n_{i} - t + 1} \right)$ is the number of possible window shifts. Then these were averaged over all $n = 24$ participants:

$$MSE\left( t \right) = \frac{1}{24}\mathop \sum \limits_{i = 1}^{24} MSE\left( {i, t} \right).$$

This averaging procedure ensured that, although some participants did not have enough data for 20 window shifts, their errors were weighted equally.

We then calculated the overall mean squared error (MSE) over all 10 values of $t$:

$$MSE_{overall} = \frac{1}{10}\mathop \sum \limits_{{t \in \left\{ {5, \ldots ,50} \right\}}} MSE\left( t \right).$$

As before, $MSE_{overall}$ weights errors equally for all participants and all values of $t$. To calculate errors for the *training* set, we followed the same procedure as for test errors $MSE\left( {i,t} \right)$, except we also summed over the $t$ observations in the partial time series (window $k$) used in training the model:

$$MSE_{train} \left( {i,t} \right) = \frac{1}{{N_{i,t} }}\mathop \sum \limits_{k = 1}^{{N_{i,t} }} \mathop \sum \limits_{j = 1}^{t} \left( {y_{itkj} - \hat{y}_{itkj} } \right).$$

Finally, to avoid over-penalizing large prediction errors, we considered an alternative error metric where the squared error $MSE\left( {i,t} \right)$ was replaced by the median absolute error (MAE):

$${\text{MAE}}\left( {i,t} \right) = {\text{median}}\left\{ {\left| {y_{itk} - \hat{y}_{itk} } \right|} \right\}_{k = 1}^{{N_{i,t} }} ,$$

which is less sensitive to timepoints with large discrepancies between the predicted and observed LRM-50.

Other models, such as multivariate linear regression, time series regression (using the *dyn* R package), and generalized additive models (using the *gamlss* R package) were considered at this stage, but did not improve performance or goodness-of-fit of the final ensemble.

### Variable selection

To identify the most important subset of variables for predicting LRM-50, we quantified a variable's importance by the average increase in MSE (%IncMSE) when permuting that variable within a random forest model. We also considered importance based on the increase in node purity, which is measured by the Gini index. This data-driven framework for feature selection has been previously deployed in behavioral contexts, including the identification of self-assessed and imaging biomarkers of cognitive impairment^44,45^. Through Monte Carlo sampling of 50% of the data, we obtained 100 rankings of variable performance. The most important variables were then defined as those that appeared in the top 10 with the highest frequency (Fig. S4). While this metric ("Top 10 Rate") identifies variables that are consistently important to prediction, those scoring lower are not necessarily uninformative. In statistical analyses, we considered both models fit on the full set of predictors, as well as the subset consisting of the most important variables.

### Shiny application

**Figure 4:**
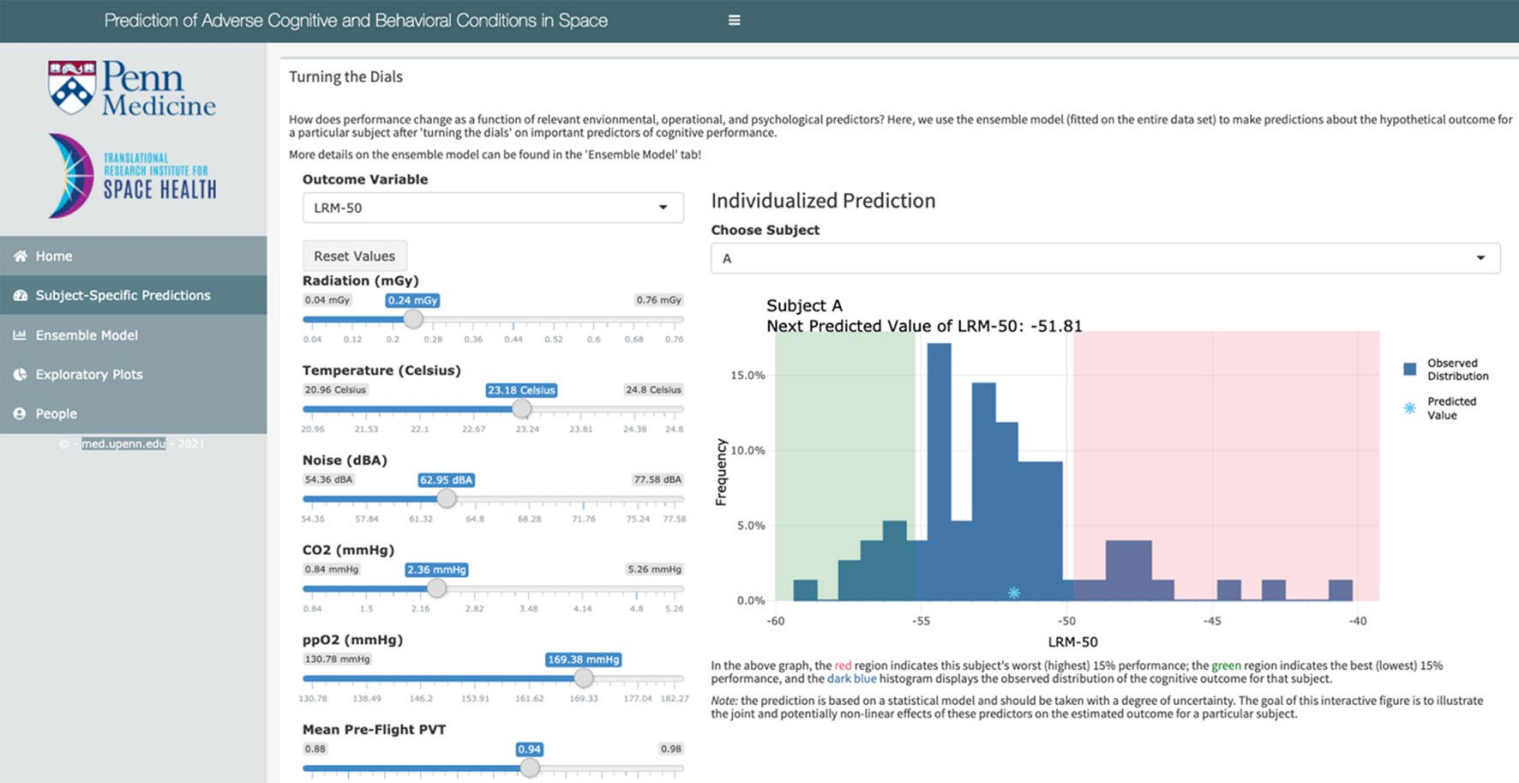
A screenshot of the R Shiny application implementing the ensemble prediction model. In the left panel, the user may "toggle" the value of each predictor (pre-set to averages observed for the individual astronaut). In the right panel, the resulting individualized predicted LRM-50 score for the selected participant is displayed (blue star at bottom of graph), along with the distribution of that astronaut's observed scores over the entire in-flight period. The prediction was made given the most up-to-date information for the astronaut, and the red and green regions correspond to that astronaut's worst 15% and best 15% scores overall.

The ensemble model was implemented as a user-friendly and interactive R Shiny application (Fig. 4). Given the data and the fitted ensemble model, the application displays individualized LRM-50 predictions in the context of their entire performance history, and other model diagnostic information. To encourage hypothesis generation, the value of predictor variables can also be "toggled," allowing the user to view how the predicted LRM-50 changes under hypothetical sets of conditions. Finally, the application includes each participant's entire trajectory of predicted and observed LRM-50 scores (not shown).

## Results

### Importance ranking of predictors of LRM-50

**Figure 5:**
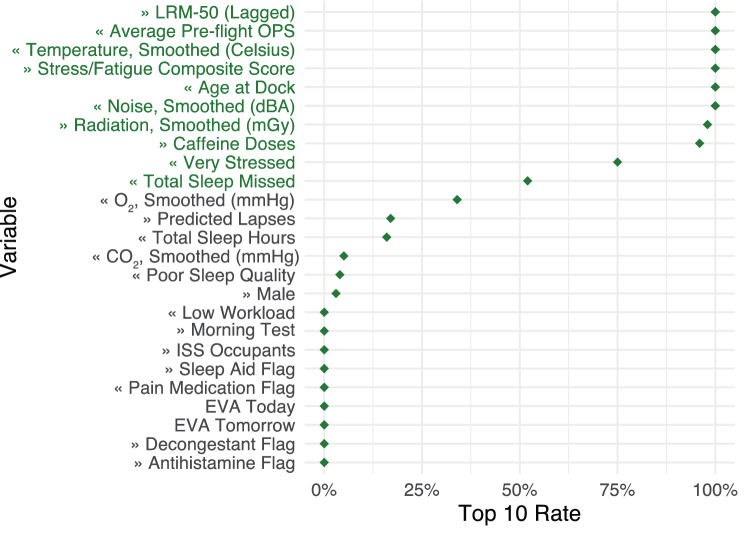
A variable's importance was measured by the increase in mean squared error (MSE) by permuting that variable in a random forest model. Variable importance rankings were obtained from 100 resampling draws. The resulting "Top 10 Rate" (x-axis) describes how a given variable, over resampling trials, is repeatedly among the 10 most important variables in a random forest model. We then defined the Top 10 variables as those which scored higher on this metric; these consisted of the lagged LRM-50 through Total Sleep Missed (green text). Arrows next to names refer to the direction of association in the linear mixed effects model (Table S8): " <  < " represents association with lower LRM-50 (better performance) and vice versa for " >  > ". Due to low counts, the EVA variables were not included in the linear mixed effects model. (OPS = Overall Performance Score; RST = Reaction Self-Test; ISS = International Space Station; EVA = extravehicular activity).

According to the random forest importance ranking, the most important predictors of LRM-50 were individual characteristics including age and average pre-flight OPS; the most recent LRM-50 score (lagged LRM-50); psychological factors including the composite stress/fatigue score; caffeine intake; total sleep missed during the most recent sleep opportunity (i.e., the sum of time taken to fall asleep, time spent awake during the night due to sleep disturbances, and time spent in bed before getting up); and smoothed environmental measurements, including temperature, noise, and radiation dose (Fig. 5). Additionally, the first 10 variables (lagged LRM-50 through total sleep missed) had a relatively high Top 10 Rate compared to the other predictors, signifying that the rankings were stable from subsample to subsample.

Other sleep variables (sleep quality and sleep duration), O_2_ and CO_2_ levels, and sex were moderately important. The test track (morning or evening RST), medication use, ISS occupancy, scheduled EVAs, and workload were rated lower, meaning that they were not consistently among the 10 most important predictors of LRM-50. Variable rankings based on node purity (Fig. S5) were similar to those noted in Fig. 5, though node purity-based importance tended to be more stable across Monte Carlo iterations.

### Prediction accuracy of the ensemble model

**Figure 6:**
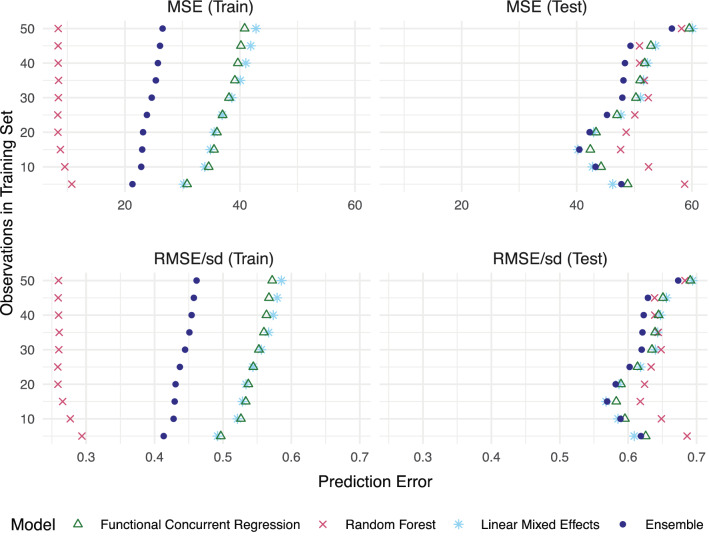
Prediction accuracy among each of the component models and the ensemble. Model performance was measured using the mean squared error (MSE) in predicting LRM-50, described in “Model validation”. A standardized measure that can be used to compare prediction accuracy for outcome data with different sizes and magnitudes was obtained by dividing the root MSE (RMSE) by the standard deviation (sd) of the outcome. The RMSE/sd represents the ratio of the model error to the overall variation of the outcome observed in the data.

Our analyses indicate that the ensemble model performed better than any single model alone over various training lengths $t$ after forward-chaining cross-validation (Fig. 6). In testing data, the ensemble model achieved the lowest MSE on average (Table S6); it out-performed all 3 of its constituent models for $t \ge 20$, but was out-performed by the LME for shorter training lengths $t < 20$. In the training data, the random forest model achieved the lowest MSE for all values of $t$.

Many of the largest MSE values were due to "spikes" in a participant's LRM-50 score that were unanticipated by all of the models (Fig. S6). In terms of the average MAE-based error, which is more robust to large prediction errors from spikes, the ensemble model continued to outperform the other models. However, its lead over the other models was less pronounced (Fig. S7).

While the previous results were based on the models’ fit on the full set of covariates, we also asked if performance was preserved if the model was fit on a subset. This subset of "Top 10" variables was determined by the variable importance ranking in “Importance ranking of predictors of LRM-50” section: they were the lagged LRM-50, noise, temperature, stress/fatigue composite, pre-flight OPS, age, caffeine doses, radiation, "Very Stressed," and total sleep missed. We also considered the models’ fit on the Top 10 variables excluding noise (due to its high interpolation rate) and including CO_2_ and O_2_ (which were environmental factors of interest and ranked highly in terms of node purity-based importance). This defined 4 additional models (Table S6), which all performed similarly, though models excluding the noise variable achieved the highest errors.

Modelling the outcome as a standardized LRM-50 score, scaled by each participant’s average and standard deviation, led to similar performance as before, with the ensemble again outperforming its components (Fig. S8). Slight improvements in performance of the ensemble and functional regression models were achieved when replacing the LRM-50 score with the standardized version.

Finally, prediction accuracy was comparable when predicting more than 1 observation in the future. In addition to varying training length $t$, we also varied the length of the test set (i.e., the number of future observations to predict in each cross-validation loop), up to 7 observations. Because the RST was administered twice a day every 4 days, this corresponded to an average chronological horizon of 15.32 days ahead. Overall, prediction accuracy was stable as the horizon grew, with marginal increases in MSE for predictions further out (Table S7).

### Nonlinear and time-varying associations between environmental factors and alertness

**Figure 7:**
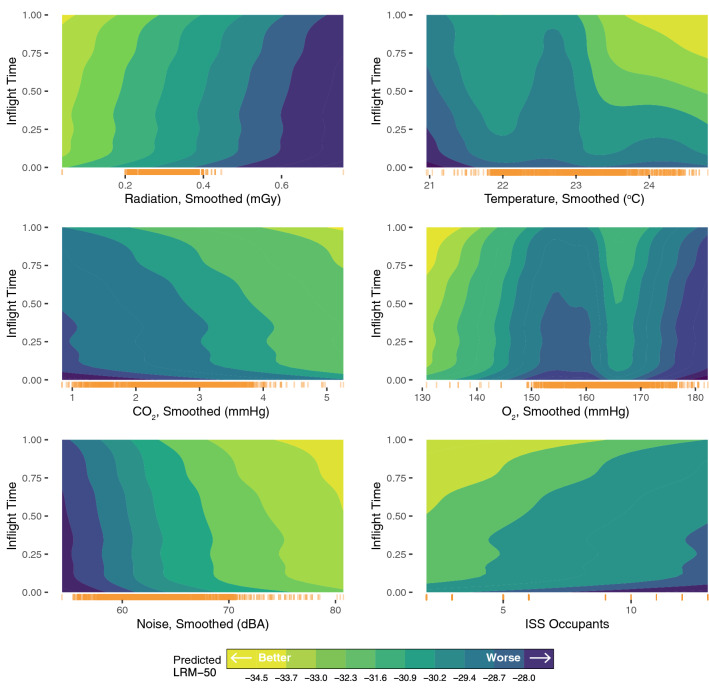
Using predictions from the functional concurrent model, these heat maps show how the non-linear effects of environmental variables on LRM-50 vary over time in mission (ranging from 0 to 1 representing the proportion of mission time elapsed). The same functional concurrent model, which was fit on the entire observed data, was used for each panel. For each environmental variable, predictions were made at a regular grid of time points between 0 and 1, and at all observed values of that environmental variable. All other variables were held at their average (continuous) or reference (categorical) value. The marginal distribution of the environmental variable observations is displayed as a rug plot (orange lines) above the x-axis. We find that better predicted performance (i.e., lower LRM-50, indicated by lighter yellow regions) is generally associated with lower radiation dose, moderate to higher temperatures, higher CO_2_, and moderate and lower O_2_.

To explore the modeled relationships between environmental conditions and LRM-50, we examined the corresponding LME coefficients (Table S8) and functional concurrent regression heat maps (Fig. 7). In both models, we found that better predicted outcomes (i.e., lower LRM-50) was associated with lower radiation dose, higher CO_2_ exposure levels, and fewer ISS occupants. Noise levels appeared to be associated with better performance; however, the majority of noise observations were imputed, and we refrain from interpreting this finding.

While the LME estimated positive effects of increased O_2_ and temperature on predicted LRM-50, the functional model revealed that these associations may be non-monotonic, suggesting that values either higher or lower than a certain range can affect vigilant attention negatively. In particular, partial pressures of O_2_ between 160 and 170 mmHg were linked to better predicted performance. The model predictions also suggest better PVT-B performance at low partial pressures of O_2_ (< 140 mmHg). Temperatures between 21.5 and 22.5 °C and higher than 23 °C were associated with better predicted performance.

In addition to the concurrent radiation levels, we calculated cumulative radiation doses for each person on each day of their mission, defined as the cumulative sum of daily exposure values in mGy. While cumulative radiation dose was found to be an important predictor for LRM-50 on top of concurrent radiation (Supplementary Methods 1), its inclusion did not ultimately improve model performance even when limited to variables ranked high in importance (Table S9).

### Individualized predictions

**Figure 8:**
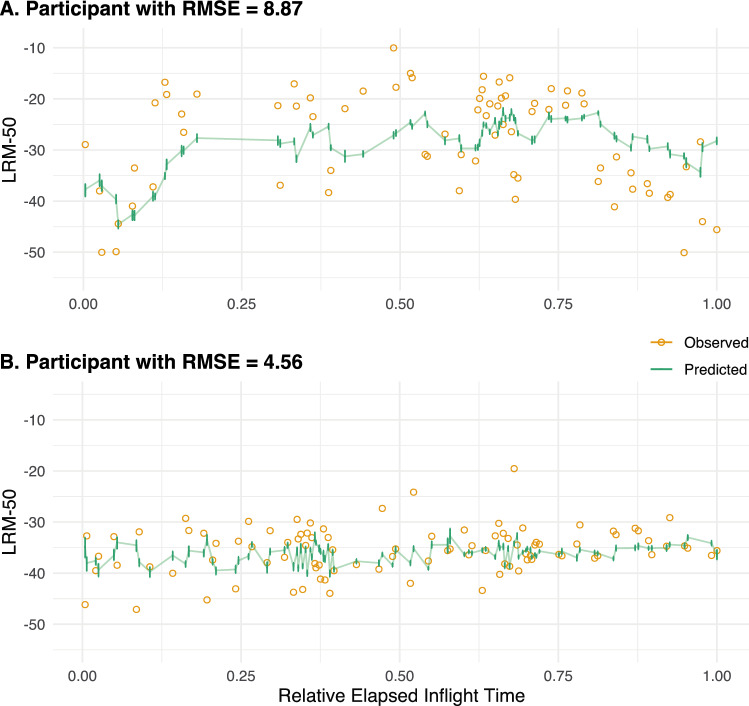
At each time point and for a given astronaut, LRM-50 can be predicted by fitting the model on all preceding data from that astronaut and the full data from other astronauts. The prediction (solid green line) is then made using covariate values from that time point. Sampling variability, given by the height of the error bars at each predicted value, was estimated as the interquartile range of predicted values from 100 bootstrap samples. In order to preserve time-varying trends, individuals were bootstrapped. The actual values of LRM-50 are displayed as hollow yellow circles. To protect astronauts' privacy, the x-axis is the proportion of elapsed mission time rather than calendar time. (A) A participant with prediction error in the highest (worst) 25th percentile (Root mean squared error (RMSE) = 8.87). (B) A participant with prediction error in the lowest (best) 25th percentile (RMSE = 4.56).

An astronaut's LRM-50 score can be predicted at an arbitrary number of future time points, but this requires knowledge of environmental conditions and other covariates at those time points. In practice, we may obtain the best prediction at a particular time point by re-fitting the model on all previous data from that individual, as well as all data collected from other participants. After fitting the model, predictions are then made using the observed covariates from that day. By repeatedly re-fitting the model and predicting the next LRM-50 score at each observation, we are able to compare the entire sequence of the observed and predicted performance for each participant (Fig. 8). To estimate sampling variation, bootstrap confidence intervals and interquartile range can be obtained by bootstrapping participants (i.e., sampling individuals' entire time series with replacement). We bootstrapped at the level of participants in order to preserve trends across the mission. The root mean squared error (RMSE) of these "chained" predictions over time ranged from 3.93 to 12.11 among astronauts (Fig. S9).

## Discussion

The spaceflight environment is host to a plethora of psychological, operational, and environmental hazards. In this paper, we proposed an ensemble model to predict vigilant attention in astronauts over the course of a space mission. In contrast to previous methods that employed a single prediction method^25^ or ensemble^26^, ours includes a dynamic component to model time-varying covariate effects. While studies of behavioral health in space or in ground-based space analog environments have traditionally focused on a small number of stressors^7,46,47^, our method addresses the intertwined and time-dependent effects of several concurrent stressors. The resulting model flexibly and accurately predicts PVT-B performance. We also identified the most important predictors of behavioral alertness as a combination of individual traits, dynamic psychological state, and environmental conditions.

Ensembles of machine learning models are increasingly popular in human health studies due to their flexibility and accommodation of non-standard data types^48^. Our results suggest that, in settings where the goal is the prediction of a time-varying outcome given a combination of person-level and irregularly measured time series, ensembles which include a functional concurrent regression^32^ are able to capture dynamic effects in a powerful way. Furthermore, the incorporation of models with both scalar and functional random effects is useful for individualized predictions (Fig. 8).

### Model performance

The ensemble model achieved the best prediction accuracy, outperforming the random forest, LME, and functional regression models in terms of test set MSE. While the random forest had the lowest training MSE, the disparity between its performance on training and test sets suggests that this model may have overfit the data. Interestingly, for most models, the MSE increased as the length of training data $t$ increased, even though we often expect errors to decrease with more training data. One possible explanation is that, for longer training periods, the test set took place later in a person's mission, when the drivers of performance may be different. This may explain why functional regression and LME, which both assume autocorrelation between successive observations, had larger errors in the training set when training time $t$ increased. By contrast, the random forest does not consider the elapsed time in mission.

In space missions, we are often precisely interested in identifying those exceptional times when individuals are at their worst. MSE is a more useful error metric for gauging performance in this case, because it is more sensitive to extreme errors compared to MAE. In our data, higher MSEs were driven by the aforementioned large and unanticipated "spikes" in LRM-50, which tended to occur in the latter half of missions. Prediction at these spikes worsened as training length $t$ increased, which may also have contributed to the observed "C" shape when plotting MSE against $t$ (Fig. 6). These observations, together with the superior performance of the ensemble model in terms of both MSE and MAE, imply that the ensemble is a suitable and robust choice for time series in which periods of low variability are interspersed with occasional spikes.

### Variable importance

Several predictors ranking highly in importance in our analyses have been previously explored in conjunction with PVT performance, including age^49^, sleep duration and time awake^50^, ambient temperature^6,51^, and lagged PVT performance (performance history)^52,53^. We note that importance is a measure of relative predictive power within the random forest model; EVA events and medication use could have been labeled as "less important" if they did not exhibit sufficient variability in our data, despite their relevance to performance in theory. The distinction between morning and evening RSTs was found to be less important, potentially due to the lack of large differences in LRM-50 distribution by test track (Fig. S10). Any differences were likely due to late evening RST administrations and chronic partial sleep loss in astronauts; this is because vigilant attention is often relatively stable across the first 16 h of the wake period, before deteriorating quickly as a result of increasing homeostatic pressure and waning circadian promotion of alertness. Finally, collinearity between variables may have affected our results (Fig. S11): for example, poor sleep quality was positively correlated with total sleep missed, which could explain why the former did not rank higher in importance when both variables were in the model. Contrary to prior studies, caffeine was associated with worse predicted performance^54^ in the LME analysis, possibly due to its inverse correlation with sleep quality and duration, suggesting that caffeine was consumed because of insufficient sleep but was not able to counteract the neurobehavioral effects of insufficient sleep.

### Nonlinear associations of LRM-50 and environmental stressors

Through heatmaps from the functional concurrent regression, we also qualitatively investigated the non-linear effects of environmental conditions on neurobehavioral performance. We found that space radiation is associated with worse performance, which has been established in rodent studies involving a PVT analogue^55,56^, but not in humans. The mechanism underlying radiation-related damage to cognitive function is not yet understood^10,12^, and this association bears further investigation.

Although the effects of CO_2_ exposure on performance are debated^57^, we observed a beneficial effect of CO_2_ consistent with a recent report in astronaut-like individuals^58^. Temperatures of 23–25 °C were also associated with better predicted LRM-50, consistent with a previous study finding better performance at "cool" temperatures of 26 °C^51^. However, other studies have not detected an effect of temperature on alertness^59^. Also, O_2_ exposure levels showed non-linear relationships with PVT performance, where low O_2_ concentrations were related to better PVT performance, possibly due to the inverse relationship between O_2_ and CO_2_. Some of the effects of CO_2_, O_2_ and temperature may be explained by central nervous system arousing properties of these exposures once they move outside a range that can easily be accommodated by homeostatic processes (e.g., increase in respiration depth and frequency as well as arousal with high CO_2_ concentrations^60^). Finally, as most of the heatmaps indicated, the brighter regions of better LRM-50 performance were predicted to occur at later timepoints when keeping the value of environmental stressor fixed. As individual performance did not generally improve over time, we conjecture that environmental stressors were gradually less coupled with poorest performance, potentially because individuals learned to adjust to the ISS environment. It should be noted that these heatmaps are meant to be hypothesis-generating rather than confirmatory, especially since some of the regions are supported with little data.

### Applications, limitations, and future directions

Our findings have three main applications. First, the models were used to identify relevant predictors of objectively assessed alertness via PVT-B in spaceflight. Self-assessments of fatigue and stress, temperature and radiation exposure, caffeine consumption, and past PVT performance were identified as principal correlates of performance. This variable selection can inform space agencies of future areas to concentrate research and mitigation measures.

Second, a tool that can visualize relationships between two predictor variables, such as the R Shiny application (Fig. 4) and functional regression heat maps (Fig. 7), could facilitate the generation of future hypotheses that can later be empirically tested.

Third, exploration-class space missions will involve communication delays and require more crew autonomy. Self-administered tests that assess readiness-to-perform can therefore be a helpful tool in guiding astronaut operational decisions. Using the most current environmental and RST measurements, the predicted LRM-50 score could be incorporated into assessments of astronaut readiness ahead of mission critical tasks and EVAs. For new participants (i.e., individuals whose data did not inform model fitting), the predicted value would be heavily weighted on the group average. This highlights the importance of using a representative sample for model fitting. Our data, which represents one of the largest studies of neurobehavioral performance in astronauts on the ISS, would be a suitable option for making predictions in astronauts, and the R shiny application is a good first step in this direction. However, further validation and tests of astronaut acceptability are required before such a tool could be used in spaceflight.

This study also has several limitations. As the main objective of the ensemble model is to optimize prediction accuracy, the model does not provide statistical inferences on the significance of the effect of any single predictor on the outcome. The coefficients (if they are available) of each component model are not guaranteed to be consistent across models, which may limit interpretability. Second, variable importance rankings are based on permutations or splits of a single variable within a random forest model; therefore, higher-order relationships between multiple predictors and their importance were not assessed. Third, our ensemble prediction weights each model equally, as no model consistently over- or under-performed. Future work could use cross-validation to determine these weights empirically. Fourth, the placement of environmental sensors was constrained by the requirements of spaceflight, and NASA did not collect ambient light data during the study period^61^. We did not consider if the missingness of measurements was itself informative. These factors may have affected the estimated associations between environmental stressors, sleep, and neurobehavioral performance. Fifth, as noted in a recent paper^34^, RST observations and sleep–wake measurements were not collected on a daily basis, which limited the ability to measure cumulative sleep loss and assess the effect of circadian misalignments. While our model included many variables previously identified as relevant to PVT performance, there may be other environmental, socioeconomic, or contextual predictors^62^ that we are missing. However, it would be straightforward to add new predictors to the existing model. Sixth, the PVT assesses a single cognitive performance domain. While vigilant attention is a prerequisite for many real-world tasks, our findings do not necessarily translate to more complex cognitive or operational tasks. Finally, we did not externally validate model performance on a new group of astronauts, and further work is needed to validate neurobehavioral assessments in spaceflight.

Our statistical methodology has natural extensions. For instance, the ensemble model uses the entire data and concurrent measurements to predict LRM-50. When new observations are made, the entire model must be fit again on the expanded data. An interesting extension could involve Bayesian updating similar to those developed for the unified model of performance^63^. In addition, our model only assesses the direct effects of environmental, psychological, and operational stressors on LRM-50; however, it would also be useful to understand the indirect effects through intermediate variables such as sleep quality and duration. Future work could involve more sophisticated functional mediation analyses that model these relationships explicitly^64^. Finally, stressors such as space radiation^65^ and sleep loss^66^ may have smaller day-to-day effects on neurobehavioral performance, but significant cumulative effects. While we considered a simple analysis of cumulative radiation, we look forward to study designs and methods that can model the effect of concurrent exposures, as well as exposure duration and history.

## Conclusions

To our knowledge, our model is the first investigation of the dynamic, non-linear relationships between common spaceflight stressors, astronaut demographics, and self-reported ratings of sleep and behavioral state on vigilant attention, while also providing individualized predictions of future performance.

The success of spaceflight depends on the physical and mental health of crew members. Our study, based on one of the largest datasets of astronaut neurobehavioral performance and sleep in space, has identified promising avenues in modelling dynamic and personalized profiles of neurobehavioral alertness. Such tools could have important implications for safety and decision-making in one of the most high-profile and dangerous occupations.

## Supplementary Information

Supplementary Information.

## Data Availability

Per funding agency requirements, the RST data analyzed in this study were uploaded to NASA's Life Sciences Data Archive (LSDA; https://lsda.jsc.nasa.gov) and, together with the environmental data, are available upon request from NASA.

## Code availability

All R code required to replicate the analyses herein and produce the R Shiny application are available at https://github.com/danni-tu/TRISH_dynamic_prediction.
