# Supplementary material for: Dynamic ensemble prediction of cognitive performance in spaceflight
Source: Sci Rep. 2022 Jun 30;12:11032. doi: 10.1038/s41598-022-14456-8 (PMC9246897; doi:10.1038/s41598-022-14456-8)
Supplement: Supplementary file 1 — Supplementary Information. [file 41598_2022_14456_MOESM1_ESM.pdf]

Dynamic Ensemble Prediction of Cognitive Performance in Spaceflight

Danni Tu [a*], Mathias Basner [b*], Michael G. Smith [b], E. Spencer Williams [c], Valerie E. Ryder [c], Amelia A. Romoser [d], Adrian Ecker [b], Daniel Aeschbach [e,f], Alexander C. Stahn [b], Christopher W. Jones [b], Kia Howard [b], Marc Kaizi-Lutu [b], David F. Dinges [b], Haochang Shou [a+]

[a] Department of Biostatistics, Epidemiology, and Informatics, University of Pennsylvania Perelman School of Medicine, Philadelphia, PA, USA

[b] Unit for Experimental Psychiatry, Division of Sleep and Chronobiology, Department of Psychiatry, University of Pennsylvania Perelman School of Medicine, Philadelphia, PA, USA

[c] Toxicology and Environmental Chemistry, National Aeronautics and Space Administration, Houston, TX, USA

[d] Center for Toxicology and Environmental Health LLC, Houston, TX, USA

[e] Department of Sleep and Human Factors Research, Institute of Aerospace Medicine, German Aerospace Center, Cologne, Germany

[f] Institute of Experimental Epileptology and Cognition Research, Faculty of Medicine, University of Bonn, Bonn, Germany

[*]Equal contribution
[+]Corresponding author at: 219 Blockley Hall, 423 Guardian Drive, University of Pennsylvania Perelman School of Medicine, Philadelphia, PA, USA

Email addresses: danni.tu@pennmedicine.upenn.edu (D. Tu), basner@pennmedicine.upenn.edu (M. Basner), hshou@pennmedicine.upenn.edu (H. Shou)

Supporting Tables and Figures

Table S1. All predictors used in the prediction models. (OPS = overall performance score).

Source	*Category*	*Variables*	*Data Type*	*Value*
Reaction Self-Test (RST)	PVT Performance	LRM-50	Time-Varying	Numeric
		Standardized LRM-50	Time-Varying	Numeric
	RST Track Type	Morning/Evening RST	Time-Varying	Binary
		Predicted Lapses	Time-Varying	Numeric
	Sleep	Total Sleep Hours	Time-Varying	Numeric
		Total Sleep Missed (Hours)	Time-Varying	Numeric
	Self-Report	Very Stressed	Time-Varying	Numeric
		Low Workload	Time-Varying	Numeric
		Poor Sleep Quality	Time-Varying	Numeric
		Stress/Fatigue Composite Score	Time-Varying	Numeric
	Medications	Caffeine Doses	Time-Varying	Numeric
		Sleep Aid Flag	Time-Varying	Binary
		Decongestant Flag	Time-Varying	Binary
		Antihistamine Flag	Time-Varying	Binary
		Pain Medication Flag	Time-Varying	Binary
Demographics		Age at Docking	Scalar	Numeric
		Sex	Scalar	Binary
		Average Pre-Flight OPS	Scalar	Numeric
Environmental		Radiation (mGy)	Time-Varying	Numeric
		Temperature (°C)	Time-Varying	Numeric
		Noise (dBA)	Time-Varying	Numeric
		CO_2 (mmHg)	Time-Varying	Numeric
		O_2 (mmHg)	Time-Varying	Numeric
		ISS Occupancy (Count)	Time-Varying	Numeric

Table S2. During the Reaction Self-Test (RST), astronauts rated their behavioral state using 11-point Likert-type rating scales, with prompts and anchors shown below. The first question varied slightly depending on the RST track type (morning or evening).

Post-Sleep (Morning RST) Scales	
1. What was the quality of your sleep?	Anchors: Good .... Poor
2. How are you feeling right now?	Anchors: Tired .... Fresh, ready to go
3. How are you feeling right now?	Anchors: Mentally sharp ... Mentally fatigued
4. How are you feeling right now?	Anchors: Energetic .... Physically exhausted
5. How are you feeling right now?	Anchors: Not stressed .... Very stressed
6. How are you feeling right now?	Anchors: Not sleepy .... Very sleepy
Pre-Sleep (Evening RST) Scales	
1. What was today's workload?	Anchors: Very High .... Very Low
2. How are you feeling right now?	Anchors: Tired .... Fresh, ready to go
3. How are you feeling right now?	Anchors: Mentally sharp ... Mentally fatigued
4. How are you feeling right now?	Anchors: Energetic .... Physically exhausted
5. How are you feeling right now?	Anchors: Not stressed .... Very stressed
6. How are you feeling right now?	Anchors: Not sleepy .... Very sleepy

Table S3. Principal components analysis (PCA) of the Self-Report 11-point ratings and their loadings on the first principal component. The loadings were used as weights to calculate a composite variable called the stress/fatigue composite score. Based on these weights, higher values of the composite score correspond to greater feelings of stress, physical and mental tiredness and fatigue, and feelings of sleepiness. Due to its low correlation with other variables, the workload was not highly weighted in this score.

Variable	***Loading***
Workload (0 = high, 10 = low)	-0.0449
Sleep Quality (0 = good, 10 = poor)	0.2295
Feeling Sleepy (0 = not at all, 10 = very much)	0.4618
Physically Exhausted (0 = energetic, 10 = physically exhausted)	0.4898
Mentally Fatigued (0 = mentally sharp, 10 = mentally fatigued)	0.4607
Tiredness (0 = tired, 10 = fresh, ready to go)	-0.4812
Stress (0 = not stressed, 10 = very stressed)	0.2198

Table S4. To match RST observations to corresponding values of environmental and operational variables, we first summarized variables in terms of their daily value (if the variables were measured daily) or hourly average (if measured more frequently). When temperature and noise data were collected at multiple sensors simultaneously, efforts were made to "location match" the data when possible, by using the sensor measurement in the same module where the RST was taken (see Section 2.6). LOESS interpolation was used for radiation, temperature, CO_2, and O_2. Linear interpolation was used for noise.

Variable	*Frequency of Collected Measurements*	*Value Used with RST Observation*	*Location Matching (if available)*	*Interpolated Value*
Radiation (mGy)	Daily	Daily value	No	LOESS
Temperature (°C)	Multiple times per minute	Hourly average	Yes	LOESS
Noise (dBA)	Once per minute	Hourly energetic average	Yes	Linear
CO_2 (mmHg)	Multiple times per minute	Hourly average	No	LOESS
O_2 (mmHg)	Multiple times per minute	Hourly average	No	LOESS
ISS Occupancy (Count)	Daily	Daily value	N/A	N/A

Table S5. Rates of missingness for time-varying variables. The percentage of missing observations was calculated as the number of in-flight RST observations taken at times where the corresponding value of the environmental variable (i.e., the daily average of radiation or ISS occupants, and the hourly average of noise, temperature, CO_2, and O_2) was available, divided by the total number of in-flight RST observations (n = 2094). Notably, almost all values of noise were missing, since that variable was only recorded on 47 unique 24-hour measurement periods throughout the study period. Sleep diary data (i.e., bedtime, time taken to fall asleep, and duration of asleep) was only collected during the morning RST, so the proportion of missing observations was calculated out of the 1,105 in-flight morning RSTs.

Variable	**Missing Observations (%)**
Radiation Dose (mGy)	5.40
Noise (dBA)	99.71
Temperature (°C)	3.63
CO_2 (mmHg)	7.02
O_2 (mmHg)	22.97
ISS Occupants (Count)	0.00
Sleep Diary	10.68

Table S6. In testing data, the ensemble model outperforms all component models in terms of averaged mean squared error (MSE) (see Section 2.8 for details). Values in parentheses represent the interquartile range of 25th and 75th percentiles. The model trained on the full set of covariates ("All") performed best, but performance was similar when retaining only the Top 10 variables (Section 3.1). Including the O_2, CO_2, and noise variables did not drastically alter performance, although models excluding noise performed the worst overall.

Covariates	Including Noise Variable	Variable	Linear Mixed Effects	Random Forest	Functional Concurrent Regression	Ensemble
**All**	Yes	MSE (Test)	48.86 (43.77, 52.11)	52.18 (50.3, 52.47)	49.13 (44.92, 51.61)	**46.92 (43.76, 48.31)**
**Top 10**	Yes	MSE (Test)	49.34 (45.14, 52.51)	51.48 (49.71, 51.63)	50.28 (47.57, 52.1)	**47.60 (45.24, 49.16)**
**Top 10 + CO_2 + O_2**	Yes	MSE (Test)	49.48 (45.18, 52.52)	51.76 (50.1, 52.1)	50.20 (47.3, 52.02)	**47.52 (45.35, 49.14)**
**Top 10**	No	MSE (Test)	49.82 (46.12, 52.29)	51.06 (49.28, 50.98)	50.97 (48.6, 52.82)	**47.98 (45.67, 49.18)**
**Top 10 + CO_2 + O_2**	No	MSE (Test)	49.81 (46.02, 52.16)	51.49 (49.18, 51.75)	50.74 (48.2, 52.49)	**48.04 (45.83, 49.26)**
**All**	Yes	MSE (Train)	37.57 (35.03, 40.78)	**8.80 (8.39, 8.72)**	37.17 (35.62, 39.51)	24.26 (23.05, 25.66)
**Top 10**	Yes	MSE (Train)	38.74 (36.31, 41.85)	**9.87 (9.57, 9.75)**	39.87 (38.58, 42.1)	26.00 (24.86, 27.38)
**Top 10 + CO_2 + O_2**	Yes	MSE (Train)	38.73 (36.3, 41.82)	**9.09 (8.68, 9.04)**	39.57 (38.28, 41.8)	25.47 (24.42, 26.75)
**Top 10**	No	MSE (Train)	39.54 (37.27, 42.58)	**9.69 (9.4, 9.53)**	40.65 (39.39, 42.84)	26.36 (25.26, 27.7)
**Top 10 + CO_2 + O_2**	No	MSE (Train)	39.47 (37.2, 42.49)	**9.62 (9.31, 9.48)**	40.37 (39.1, 42.59)	26.25 (25.16, 27.55)

Table S7. Assuming that covariate values are known at future timepoints, model performance remains acceptable when predicting more than 1 timepoint ahead. Prediction performance was assessed using the same method as described in Section 2.8, but we allowed the size of the test set to range from 1 to 7 future observations. Values in parentheses represent the interquartile range of 25th and 75th percentiles. Because the RST was administered twice a day approximately every 4 days, these future predictions corresponded to predictions of around 2 to 15 days ahead on average. Cell values are the mean squared error in the testing data averaged over participants, then length of training days t. Overall, we find that the errors increase slowly as the prediction horizon grows.

Future Observations in Test Set	Average Length of Prediction Horizon (Days)	Linear Mixed Effects	Random Forest	Functional Concurrent Regression	Ensemble
**1**	1.88	48.86 (43.77, 52.11)	52.18 (50.3, 52.47)	49.13 (44.92, 51.61)	**46.92 (43.76, 48.31)**
**2**	4.75	49.86 (44.84, 52.77)	53.48 (51.76, 53.85)	50.03 (45.69, 52.13)	**47.92 (44.53, 49.05)**
**3**	6.55	50.17 (45.86, 52.91)	53.80 (51.31, 54.19)	49.95 (46.12, 51.98)	**48.03 (45.11, 49.06)**
**5**	11.34	50.86 (48.01, 53.18)	53.87 (51.8, 54.67)	49.78 (47.55, 51.73)	**48.05 (46.5, 49.47)**
**7**	15.32	51.69 (49.23, 53.88)	54.49 (53.02, 54.99)	50.31 (48.14, 52.24)	**48.57 (47.22, 49.56)**

Table S8. Coefficients for the linear mixed effects model to predict LRM-50 score, with a random intercept for each participant and AR1 correlation structure. To enable comparisons of coefficients between variables, both the numeric covariates and the outcome were *z*-scored (i.e., linearly scaled to have a mean of 0 and a standard deviation of 1). Positive coefficients are associated with an increase in LRM-50 (worse performance); negative coefficients are associated with a decrease in LRM-50 (better performance). Due to low numbers of EVAs, neither EVA flag was included in the model. A limitation of this model is that it can only accommodate linear relationships between predictors and the outcome, which may not reflect their actual relationship. Note: *$p < 0.05$; **$p < 0.01$.

Variable	**Coefficient (95% CI)**
(Intercept)	-0.305 (-1.011, 0.400)
Radiation, Smoothed (mGy)	0.011 (-0.029, 0.052)
Noise, Smoothed (dBA)	-0.090 (-0.120, -0.059)**
CO_2, Smoothed (mmHg)	-0.014 (-0.052, 0.023)
O_2, Smoothed (mmHg)	-0.001 (-0.036, 0.034)
ISS Occupants	0.018 (-0.016, 0.053)
Temperature, Smoothed (°C)	-0.061 (-0.111, -0.011)*
Sex = Male	0.305 (-0.555, 1.164)
Age at Dock	-0.082 (-0.428, 0.264)
Average Pre-flight OPS	-0.207 (-0.514, 0.100)
Sleep Aid Flag	0.058 (-0.077, 0.192)
Antihistamine Flag	0.010 (-0.017, 0.037)
Morning RST	0.107 (0.046, 0.168)**
Stress/Fatigue Composite Score	0.179 (0.143, 0.215)**
Low Workload	-0.019 (-0.050, 0.012)
Poor Sleep Quality	-0.005 (-0.046, 0.035)
Very Stressed	-0.017 (-0.065, 0.030)
Total Sleep Hours	-0.059 (-0.096, -0.023)**
Total Sleep Missed	-0.001 (-0.037, 0.034)
Predicted Lapses	0.003 (-0.038, 0.045)
Caffeine Doses	0.020 (-0.035, 0.074)
Decongestant Flag	0.104 (-0.135, 0.344)
Pain Medication Flag	-0.088 (-0.229, 0.053)
LRM-50 (Lagged)	0.121 (0.084, 0.157)**

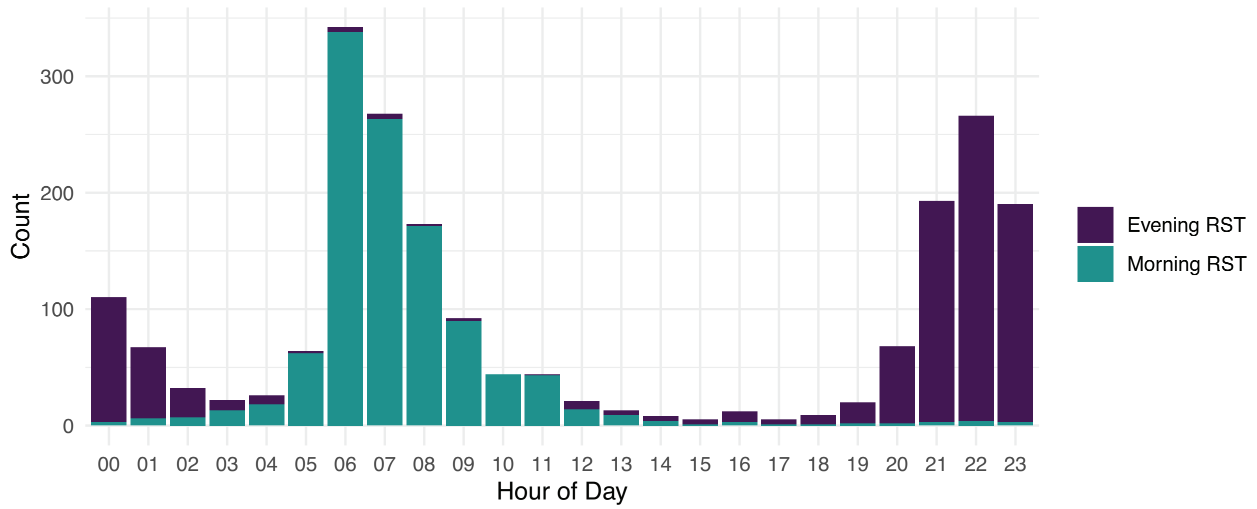

Figure S1. In-flight RST observations occurred at all hours of the day, though morning RSTs (which were taken after awakening) were concentrated in the morning hours and evening RSTs (taken before bedtime) in the nighttime hours.

A. Schematic of ISS Modules

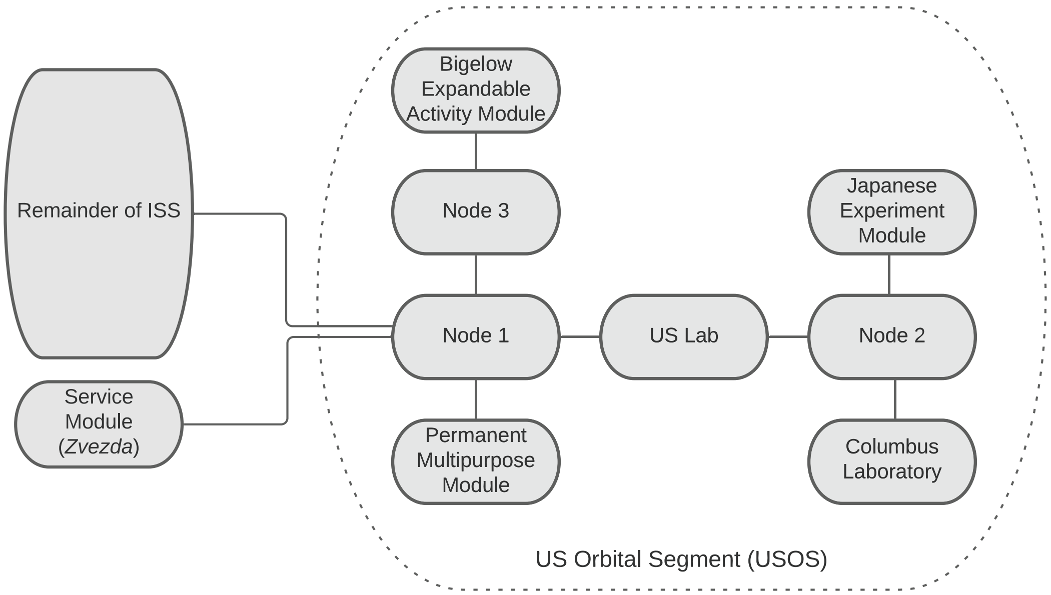

B. Locations of MCA Sample Ports Aboard ISS

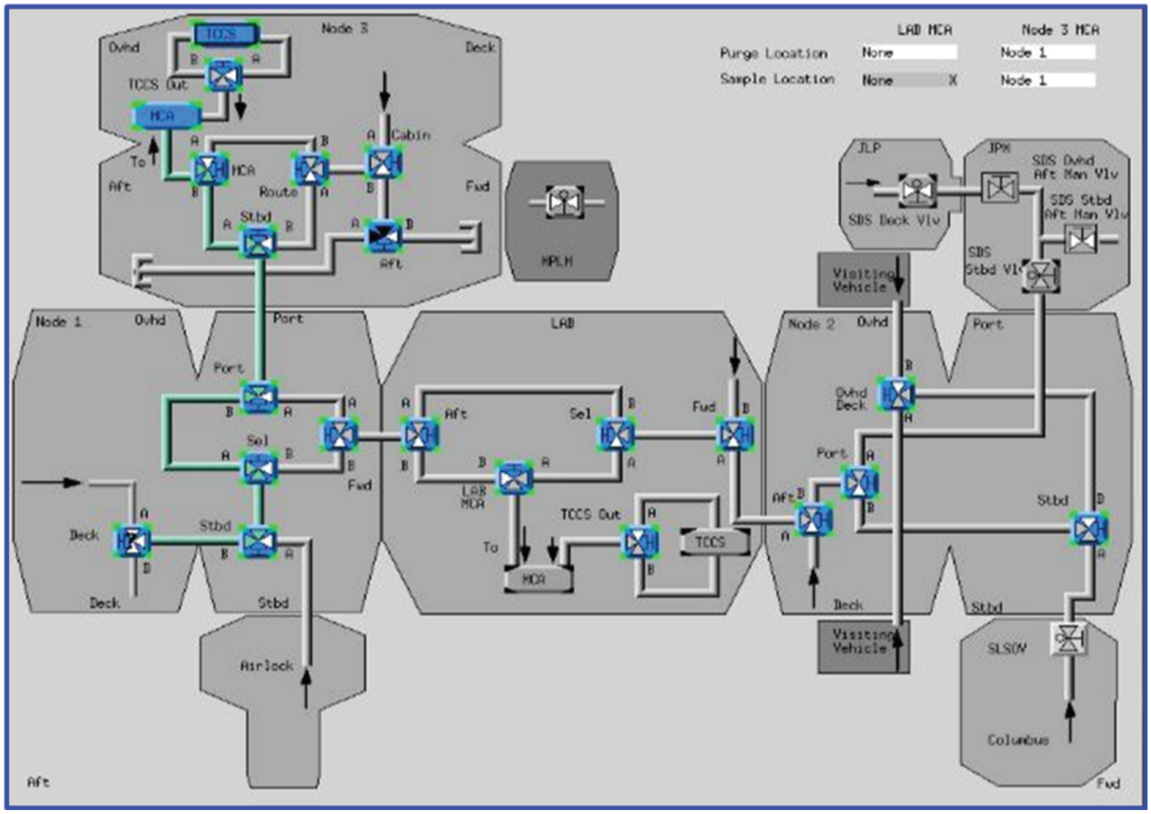

Figure S2. Modules of the International Space Station (ISS). Panel A: schematic of the ISS modules in the US Orbital Segment (USOS), where the environmental data used in this study was recorded. Most of the in-flight RST observations occurred in the US and Node 2 modules (17.4% and 75.5%, respectively). Panel B: locations of the Major Constituent Analyzer (MCA) sample ports (shown as blue squares with a blue cross and 4 green dots) throughout the USOS modules. These ports sampled CO_2 and O_2 (and other atmospheric components) into mass spectrometer-based MCA units located in Node 3 and US Lab.

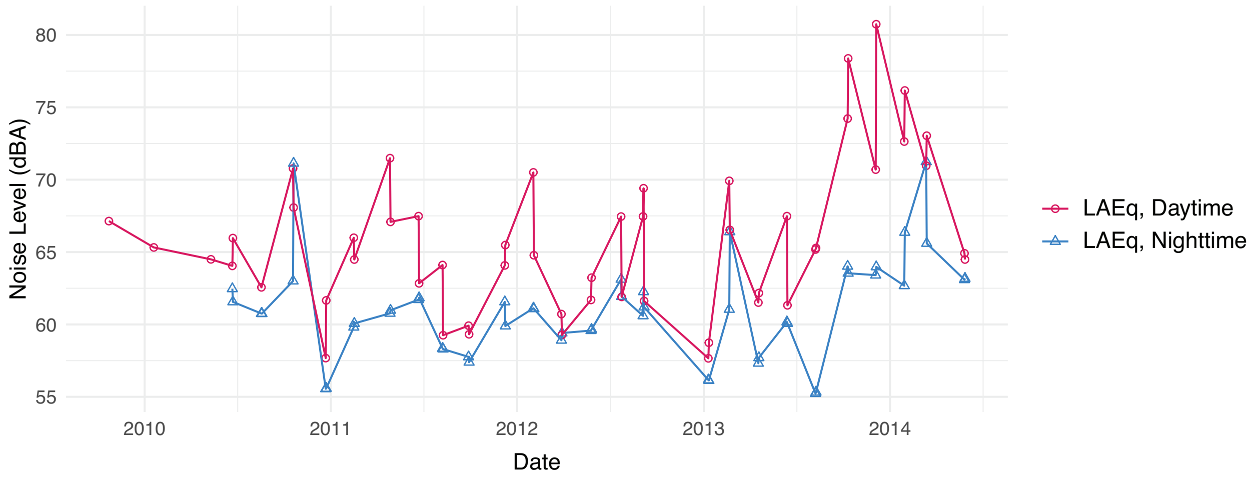

Figure S3. Average noise was higher during daytime hours (7:00 to 22:59) than nighttime hours (23:00 to 6:59). In the study period, noise levels in A-weighted decibels (dBA) were measured during 47 occasions lasting approximately 24 hours each. Red circles correspond to daytime noise measurements, averaged using the energetic average ($L_{A,eq}$). Blue triangles correspond to nighttime measurements.

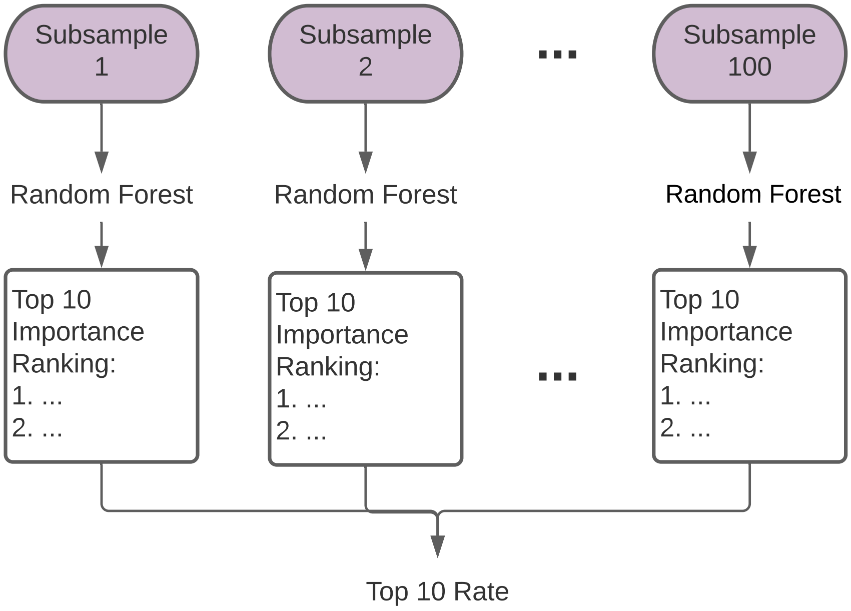

Figure S4. Variable importance defined in Section 2.9 was calculated by repeatedly fitting random forest models to resampled subsets of the data. For each subsample, the corresponding importance ranking given by the %IncMSE metric was determined. Then, the overall Top 10 Rate for each variable was calculated as the proportion of subsamples where that variable was among the 10 most important.

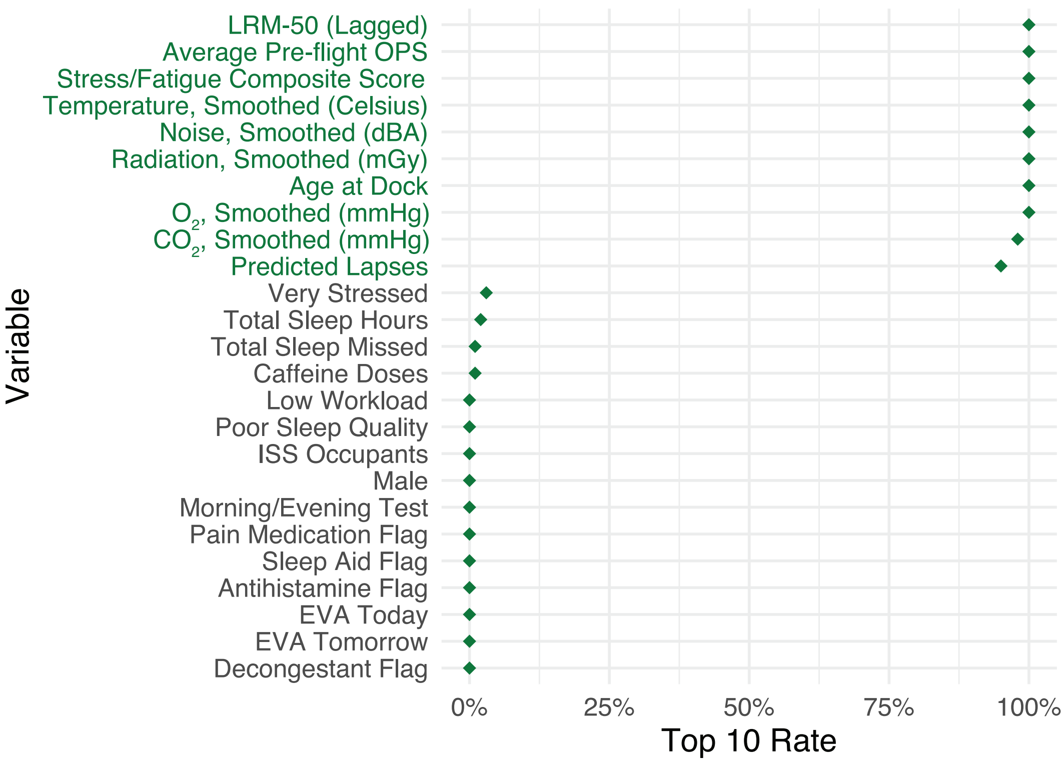

Figure S5. We also considered assessing variable importance by the decrease in node impurity, as measured by the Gini coefficient after splitting on that variable, averaged over all trees in a random forest. To understand which variables were consistently important in different subsamples of the data, variable importance rankings were obtained from 100 resampling draws. The resulting "Top 10 Rate" (x-axis) describes how a given variable, over resampling trials, is repeatedly among the 10 most important variables in a random forest model. We then defined the Top 10 variables as those which scored higher on this metric; these consisted of the lagged LRM-50 through the number of predicted lapses (green text). Compared to importance measured by the increase in MSE (Figure 5), node impurity tends to have less variability between resampling iterations. The top 7 variables in terms of node purity (lagged LRM-50 through age) were the same as those from the increase in MSE. (OPS = Overall Performance Score; RST = Reaction Self-Test; ISS = International Space Station; EVA = extravehicular activity.)

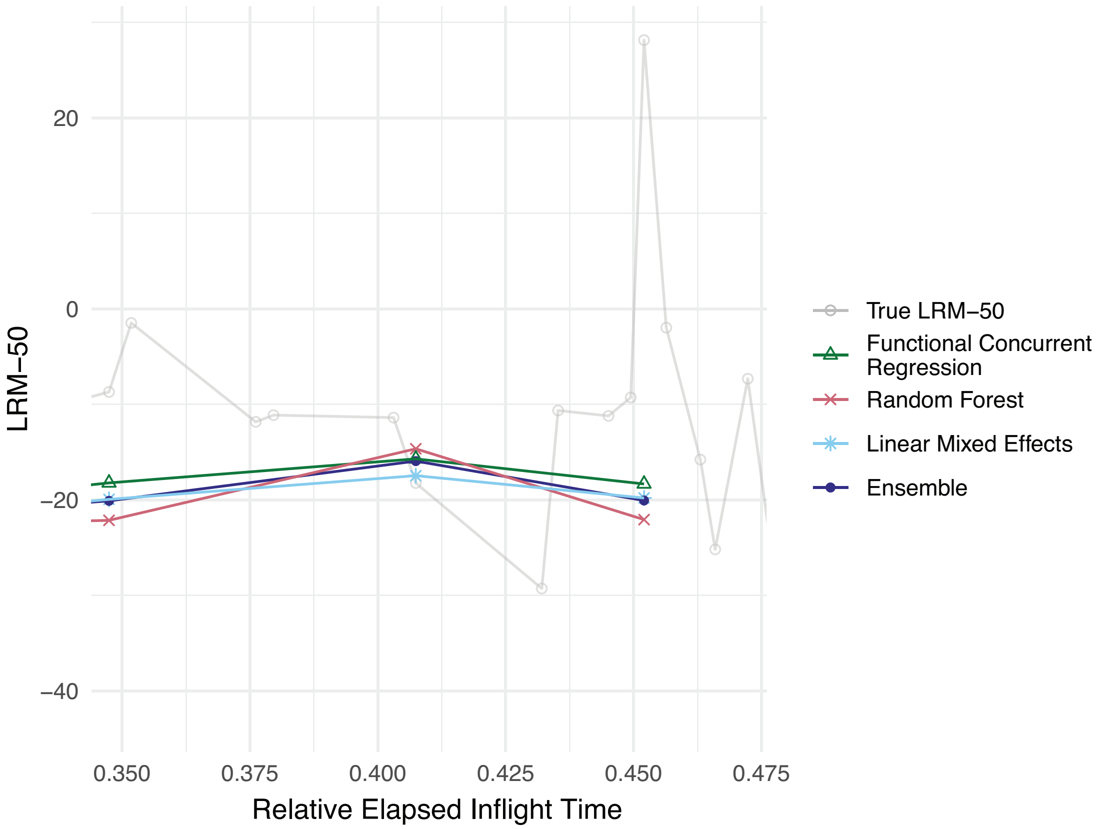

Figure S6. Example of an extreme "spike" in observed LRM-50 from one participant. The light gray hollow circles and line correspond to actual LRM-50 observations. The points and lines in color represent the model predictions at the spike and 5 or 10 observations previous. To protect astronauts' privacy, the x-axis is the proportion of elapsed mission time rather than calendar time. At the spike, which occurs just after time = 0.45, the true LRM-50 = 28.14, while more recent values ranged from -30 to -5. The models perform adequately at previous points, but are not able to anticipate the spike. This disparity between predicted values and the true value resulted in large MSEs of 2000-2500.

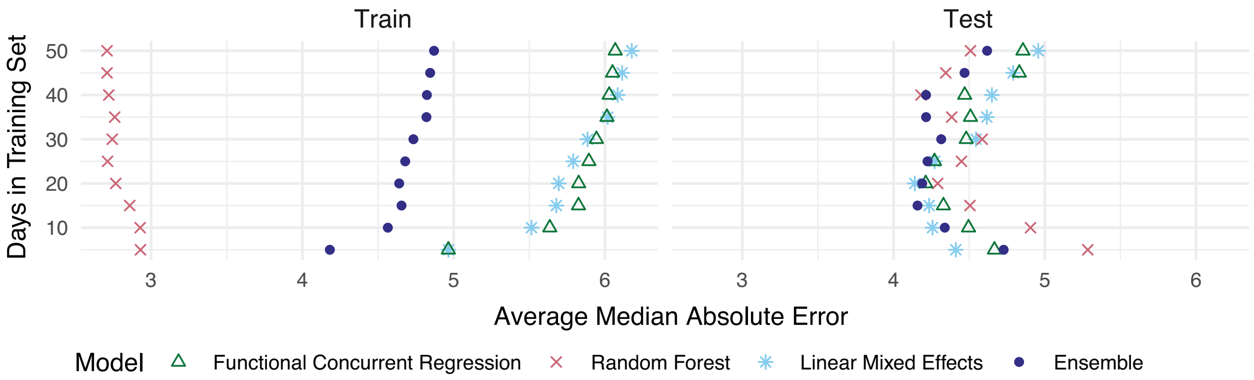

Figure S7. Because the mean squared error (MSE) tends to heavily penalize large errors in predicting LRM-50, we also compared the models in terms of the *median* absolute error, which is more robust to extreme values (i.e., by penalizing them less). The median absolute error (MAE) was calculated using a similar aggregation procedure as in Section 2.8, but replacing the $MSE(i,t)$ with $MAE(i,t)$ defined in Section 3.2. The scale of median absolute errors was smaller than that of square-root MSEs, implying that the latter were likely influenced by larger errors. The ensemble model continued to out-perform the other models on average, though its lead over other models is narrower in terms of the MAE.

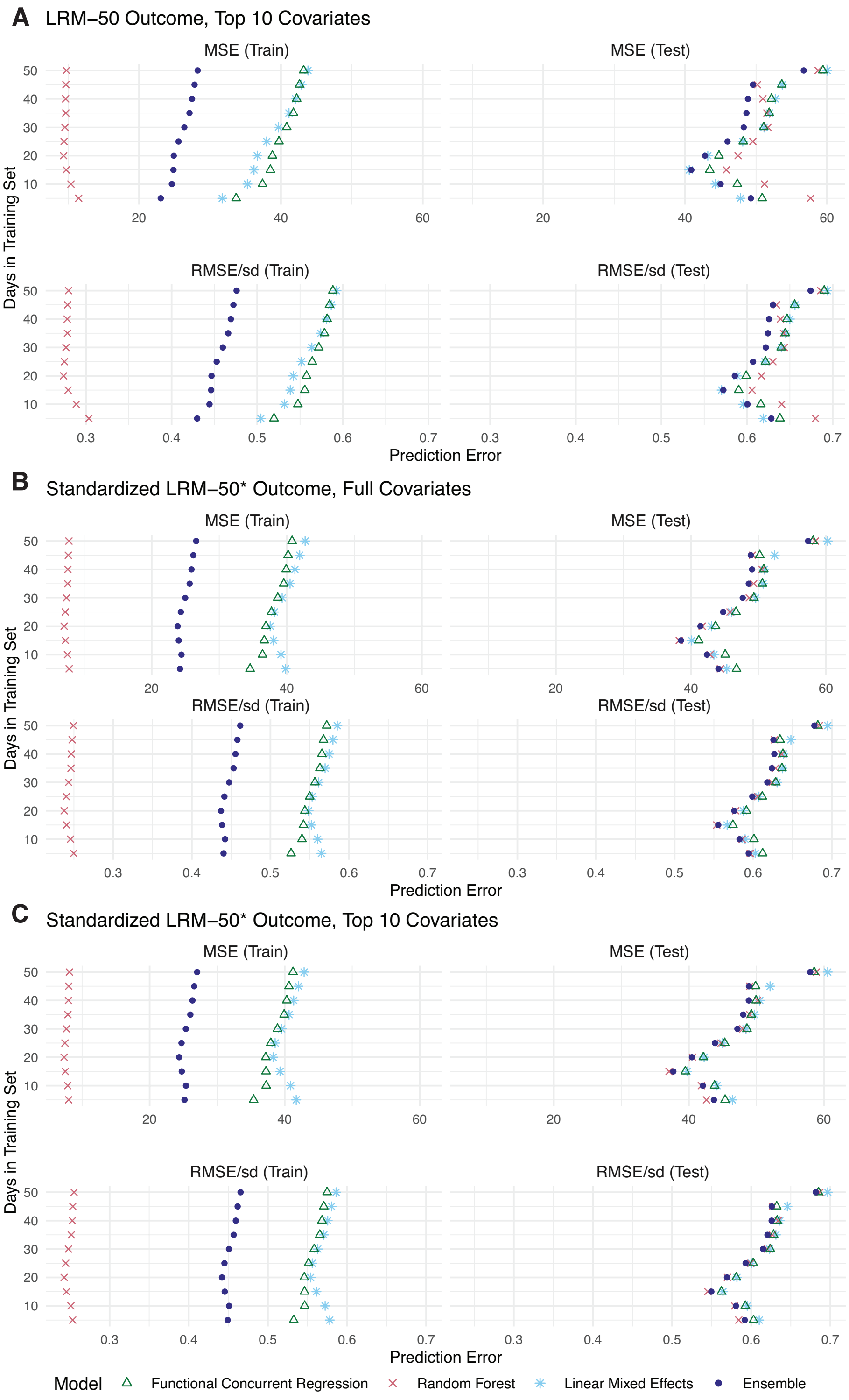

Figure S8. The ensemble model again outperformed the 3 component models under various settings. Panel A: only the top importance variables (defined by the union of the environmental variables and the 10 variables most frequently ranked in the Top 10 in variable importance; see Figure 5) were included as predictors of LRM-50. Panels B and C: we also considered the standardized LRM-50 outcome, calculated as the LRM-50 score linearly scaled by each participant's mean and standard deviation of LRM-50. *For the standardized LRM-50 outcome, the prediction errors were transformed back to the original scale to enable comparisons.

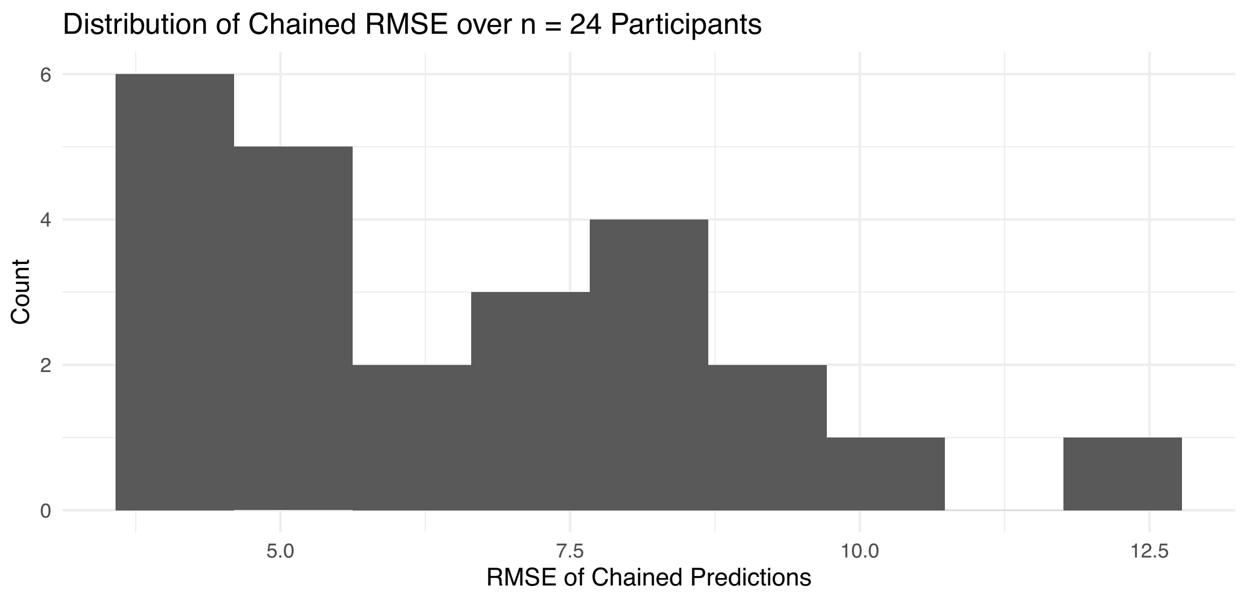

Figure S9. At any point in time, the most up-to-date information can be used to predict the next LRM-50 score, assuming that all covariate values at that future point are known. Using the procedure outlined in Section 3.4, we obtain a series of "chained" predictions for each participant. This histogram shows the distribution of the average chained RMSE among the 24 participants in our study. The mean RMSE = 6.56 (sd = 2.19) and the median RMSE = 6.12 (IQR = 4.78 to 8.02).

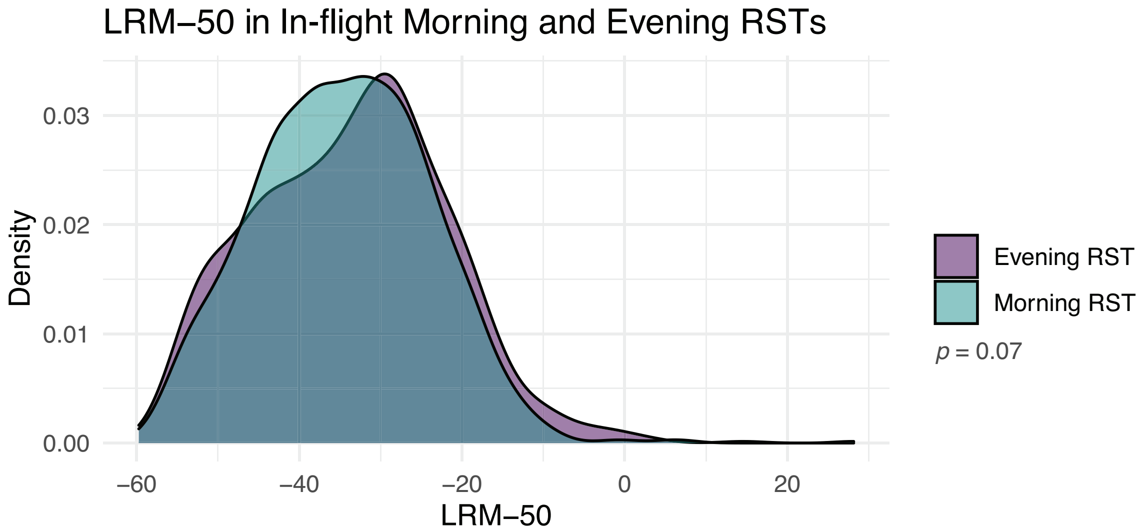

Figure S10. The distribution of LRM-50 scores in morning (n = 1105) and evening (n = 989) in-flight RST observations. The Welch two-sample *t*-test for difference in means between LRM-50 values from morning and evening tests had a *p*-value of 0.07 (*t* statistic = -1.84, *df* = 2006).

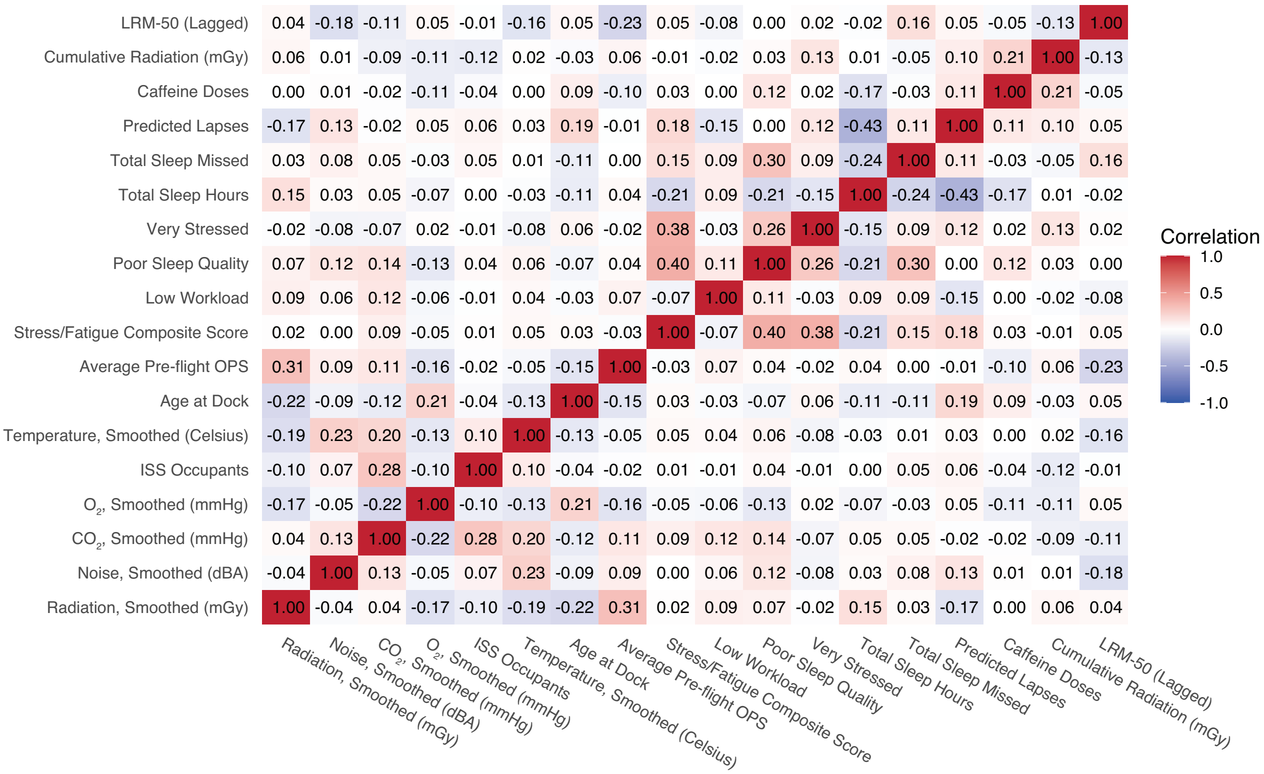

Figure S11. Heatmap of Pearson correlation between all numeric variables included in the ensemble model.

Supplementary Methods 1: Cumulative Radiation Dose

Cumulative radiation dose was calculated for each person on each in-flight day of their mission, using the cumulative sum of (smoothed) daily radiation dose in mGy.

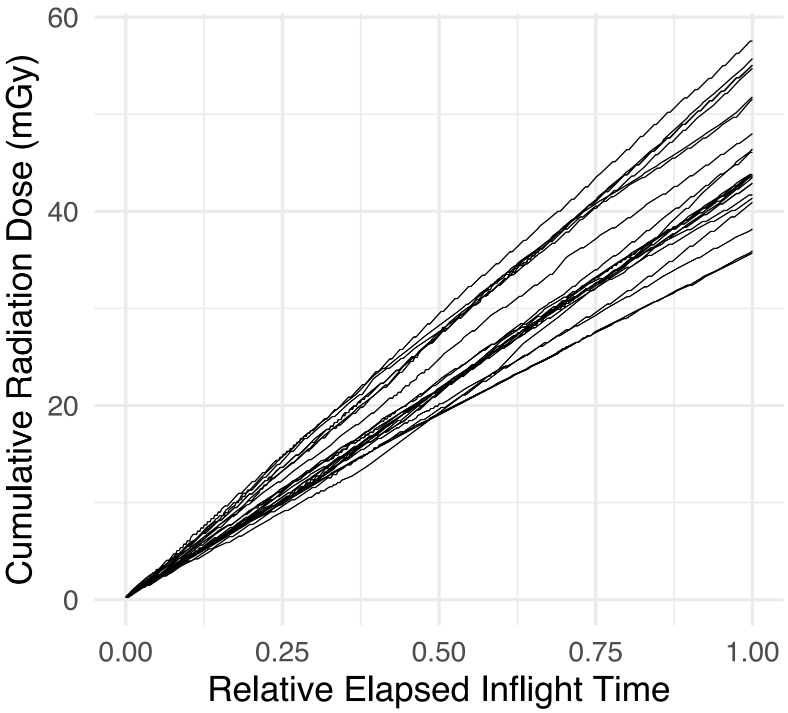

Figure S12. Cumulative radiation dose trajectories for all 24 astronauts in our data.

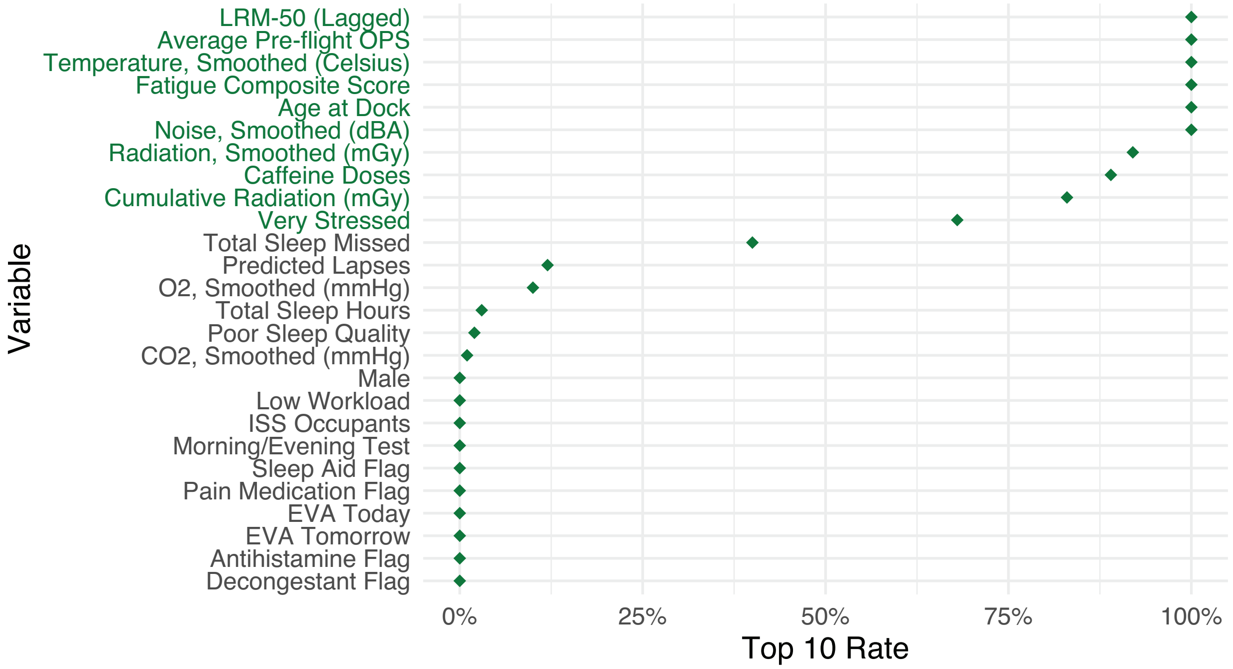

Figure S13. Random forest variable importance defined by the increase in MSE (Section 2.9) for a model including cumulative radiation (mGy). Both the concurrent radiation dose and cumulative radiation were found to be important predictors of LRM-50.

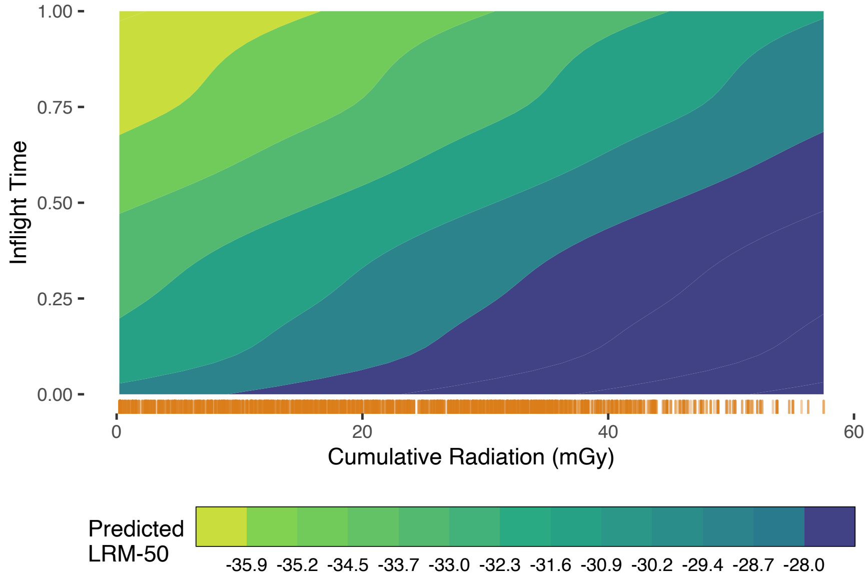

Figure S14. Using functional concurrent regression models containing cumulative radiation dose (mGy), we again produced heatmaps of the association between inflight time, cumulative radiation dose, and predicted LRM-50 (Section 3.3). The marginal distribution of the environmental variable observations is displayed as a rug plot (orange lines) above the x-axis. Similar to our findings for daily radiation dose (mGy), less cumulative radiation is associated with better predicted performance.

Table S9. Including cumulative radiation as a covariate did not dramatically alter model performance in terms of averaged mean squared error (MSE) (see Section 2.8 for details). Values in parentheses represent the interquartile range of 25th and 75th percentiles. The "Top 10" variables were defined by the 10 most important variables (Figure S13), which included cumulative radiation. As before, the model trained on the full set of covariates ("All") performed best, but performance was similar when retaining only the new set of Top 10 variables.

Covariates	Including Noise Variable	Variable	Linear Mixed Effects	Random Forest	Functional Concurrent Regression	Ensemble
**All**	Yes	MSE (Test)	48.82 (44.03, 51.83)	52.16 (50.38, 52.67)	49.37 (45.07, 52.1)	**46.98 (43.95, 48.56)**
**Top 10**	Yes	MSE (Test)	49.26 (45.09, 52.22)	51.38 (49.89, 52.2)	50.41 (47.57, 52.61)	**47.44 (45.28, 48.96)**
**Top 10 + CO_2 + O_2**	Yes	MSE (Test)	49.37 (45.13, 52.15)	51.85 (50.11, 52.27)	50.37 (47.38, 52.48)	**47.43 (45.25, 48.95)**
**Top 10**	No	MSE (Test)	49.73 (45.99, 52.11)	51.36 (49.54, 51.3)	50.48 (47.83, 52.1)	**47.74 (45.52, 48.82)**
**Top 10 + CO_2 + O_2**	No	MSE (Test)	49.59 (45.96, 52.03)	51.11 (49.28, 52.65)	50.66 (48.05, 52.55)	**47.71 (45.36, 49.16)**
**All**	Yes	MSE (Train)	37.64 (35.05, 40.93)	**8.71 (8.33, 8.61)**	36.72 (34.95, 39.26)	24.15 (22.88, 25.61)
**Top 10**	Yes	MSE (Train)	38.80 (36.31, 42)	**9.50 (9.17, 9.38)**	39.63 (38.22, 42.06)	25.71 (24.57, 27.11)
**Top 10 + CO_2 + O_2**	Yes	MSE (Train)	38.80 (36.31, 41.98)	**8.93 (8.54, 8.86)**	39.41 (37.98, 41.83)	25.31 (24.21, 26.64)
**Top 10**	No	MSE (Train)	39.54 (37.2, 42.66)	**9.35 (9.01, 9.18)**	40.12 (38.71, 42.51)	25.99 (24.86, 27.35)
**Top 10 + CO_2 + O_2**	No	MSE (Train)	39.54 (37.2, 42.66)	**9.60 (9.32, 9.48)**	40.41 (38.97, 42.83)	26.18 (25.01, 27.62)
